# Structure and biological function of ENPP6, a choline-specific glycerophosphodiester-phosphodiesterase

**DOI:** 10.1038/srep20995

**Published:** 2016-02-18

**Authors:** Junko Morita, Kuniyuki Kano, Kazuki Kato, Hiroyuki Takita, Hideki Sakagami, Yasuo Yamamoto, Emiko Mihara, Hirofumi Ueda, Takanao Sato, Hidetoshi Tokuyama, Hiroyuki Arai, Hiroaki Asou, Junichi Takagi, Ryuichiro Ishitani, Hiroshi Nishimasu, Osamu Nureki, Junken Aoki

**Affiliations:** 1 Department of Biological Sciences, Graduate School of Sciences, The University of Tokyo, 2-11-16, Yayoi, Bunkyo-ku, Tokyo, 113-0032, Japan; 2 Graduate School of Pharmaceutical Sciences, Tohoku University, 6-3, Aoba, Aramaki, Aoba-ku, Sendai, Miyagi, 980-8578, Japan; 3 Graduate School of Pharmaceutical Sciences, The University of Tokyo, 7-3-1, Hongo, Bumkyo-ku, Tokyo, 113-0033, Japan; 4 Institute for Protein Research, Osaka University, 3-2, Yamadaoka, Suita-shi, Osaka, 565-0871, Japan; 5 Center for Kampo Medicine, Keio University School of Medicine, 35, Shinanomachi, Shinjuku-ku, Tokyo, 160-0016, Japan; 6 PRESTO (Precursory Research for Embryonic Science and Technology), JST (Japan Science and Technology Agency), 4-1-8, Honcho, Kawaguchi, Saitama, 332-0012, Japan; 7 AMED (Japan Agency for Medical Research and Development)-CREST (Core Research for Evolutional Science and Technology), 1-7-1, Otemachi, Chiyoda-ku, Tokyo, 100-0004, Japan

## Abstract

Choline is an essential nutrient for all living cells and is produced extracellularly by sequential degradation of phosphatidylcholine (PC). However, little is known about how choline is produced extracellularly. Here, we report that ENPP6, a choline-specific phosphodiesterase, hydrolyzes glycerophosphocholine (GPC), a degradation product of PC, as a physiological substrate and participates in choline metabolism. ENPP6 is highly expressed in liver sinusoidal endothelial cells and developing oligodendrocytes, which actively incorporate choline and synthesize PC. ENPP6-deficient mice exhibited fatty liver and hypomyelination, well known choline-deficient phenotypes. The choline moiety of GPC was incorporated into PC in an ENPP6-dependent manner both *in vivo* and *in vitro*. The crystal structure of ENPP6 in complex with phosphocholine revealed that the choline moiety of the phosphocholine is recognized by a choline-binding pocket formed by conserved aromatic and acidic residues. The present study provides the molecular basis for ENPP6-mediated choline metabolism at atomic, cellular and tissue levels.

Choline (trimethyl-2-hydroxyl-ethylammonium) was recognized as an essential nutrient for humans by the U.S. National Academy of Sciences in 1998^1^. Prolonged (weeks to months) ingestion of a diet deficient in choline results in hepatic, renal, pancreatic, memory and growth disorders^2^. Choline and its metabolites, assure the structural integrity and signaling functions of cell membranes. Choline is predominantly used for the synthesis of essential phospholipid components of cell membranes such as phosphatidylcholine (PC) and sphingomyelin (SM)^3^. Choline is also a source of methyl groups in the diet. For example, betaine, a choline metabolite, participates in the methylation of homocysteine to form methionine. Choline directly affects cholinergic neurotransmission and lipid transport from liver. Choline is *de novo* synthesized from phosphatidylethanolamine (PE) as a form of PC, which is catalyzed by PE *N*-methyltransferase in liver cells^4^. By contrast, in other cells choline is supplied from an outside source through choline-specific transporters expressed on their plasma membrane^5^. Since the amount of choline required is much higher than that synthesized by the liver *in vivo*, choline must be supplied from dietary sources^2^.

Phospholipids such as PC or SM in the diet are digested to choline, which is then assimilated in the small intestine and transferred to the liver, where it is converted to PC^6^. PC is secreted from the liver and circulates in the blood as a form of lipoprotein, which is sequentially degraded to free choline in the circulation^7^,^8^. In blood, choline is present in the form of choline derivatives such as PC, SM, glycerophosphorylcholine (GPC) and lysophosphatidylcholine (LPC) or a free form (free choline)^9^. PC is the most abundant choline source in blood and its concentration is at the mM level. SM and LPC are also major choline-containing phospholipids in blood, and their concentrations are at the several hundred μM level^10^. In contrast, free choline and GPC are minor components and their concentrations are only at the several μM level in the blood and cerebrospinal fluids^9^. Since *de novo* synthesis of choline occurs specifically in liver cells, it is reasonable to assume that free choline used by most cells in a body originates from choline-containing phospholipids in the blood. However, little is known about how free choline is generated in the blood.

The ecto-nucleotide pyrophosphatases/phosphodiesterases (ENPPs or NPPs) family consists of seven structurally-related ecto-enzymes^11^. Based on structural and evolutionary viewpoints, ENPP family members can be divided into two main subgroups, namely ENPP1–3 and ENPP4–7. ENPP1–3 have two N-terminal somatomedin B-like domains, a central phosphodiesterase (PDE) domain and a C-terminal nuclease-like domain, whereas ENPP4–7 have only the PDE domain in common. ENPPs hydrolyze pyrophosphate or phosphodiester bonds in (di)nucleotides and phospholipids, and are involved in a range of cellular processes. For example, ENPP2 (also known as autotaxin, ATX) has lysophospholipase D activity and hydrolyzes LPC and other lysophospholipids to generate a signaling molecule, lysophosphatidic acid (LPA), which contributes to cell growth and cell motility^12^,^13^. ENPP1, ENPP3 and ENPP4 hydrolyze nucleotides such as ATP^14^, UDP-GlcNAc^15^ and diadenosine triphosphate (Ap3A)^16^, and have roles in bone mineralization, regulation of sugar chain synthesis and platelet aggregation, respectively. ENPP7 specifically hydrolyzes sphingomyelin^17^ and contributes to sphingolipid metabolism in the intestine^18^. Recently, crystal structures of ENPP1, ENPP2 and ENPP4 have revealed that the “insertion loop” in the catalytic pocket of the PDE domain determines their substrate specificities^19^,^20^,^21^.

We and others recently found that ENPP6 has *in vitro* phosphodiesterase activities towards choline-containing compounds such as GPC^22^ and O-phosphorylcholine *N*-acyl ethanolamine^23^ to produce phosphocholine. ENPP6 hydrolyzes only choline-containing lysophospholipids, such as LPC, sphingosylphosphorylcholine (SPC), platelet-activating factor (PAF) and lysoPAF, but not other lysophospholipids^22^, suggesting that ENPP6 recognizes the choline moiety of its substrates. However, its physiological substrates, biological roles and the molecular mechanism by which it recognizes the choline moiety of its substrates remain unclear.

In this study we describe the structure and function of ENPP6. We demonstrate that ENPP6 is specifically expressed in endothelial cells of the sinusoid in the liver and developing oligodendrocytes, which actively incorporate choline, and that ENPP6 hydrolyzes choline-containing compounds such as GPC, and contributes to supplying choline to these cells. Furthermore, we solved the crystal structure of ENPP6 in complex with phosphocholine at high resolution, providing the structural basis for the choline recognition mechanism by ENPP6.

## Results

### Expression of ENPP6

**Figure 1:**
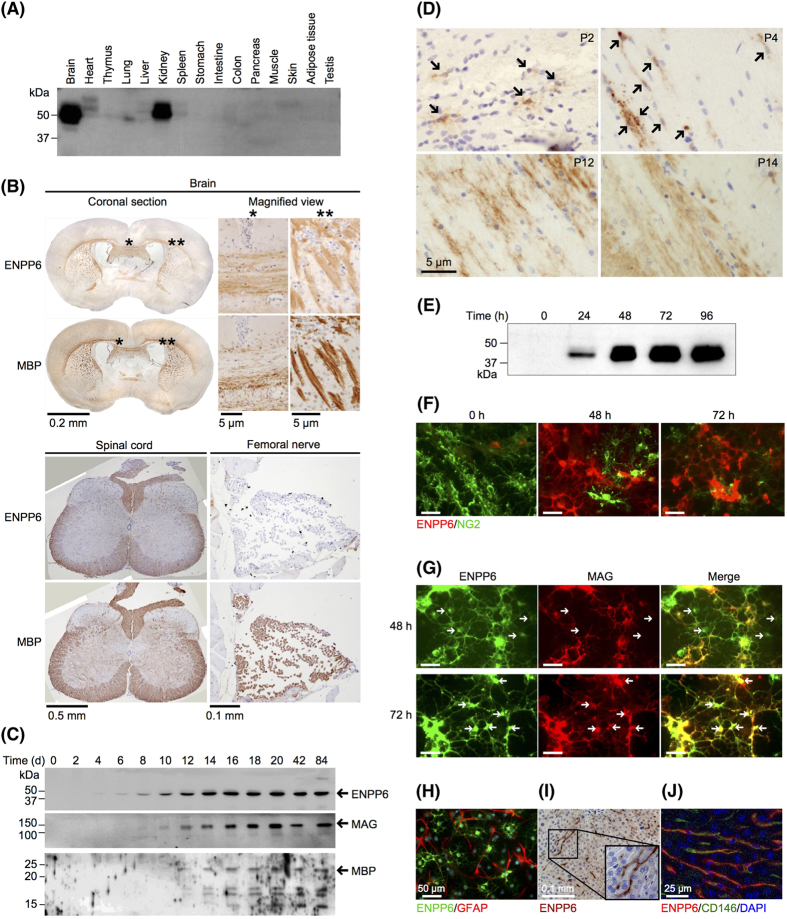
Expression of ENPP6. (**A**) Expression of ENPP6 protein in adult mice tissue. Total protein (20 μg/lane) from various mouse tissues was examined by Western blot analysis. Note that the molecular weight of ENPP6 detected in each tissue varies, possible due to the difference in glycosylation. (**B**) Immunohistochemical analysis of ENPP6 and MBP in central and peripheral nerve system in adult mice. Magnified view of the brain shows that ENPP6 was predominantly detected in the myelin sheath but not in the cell bodies of oligodendrocytes or other neural cells. (**C**) Expression of ENPP6 and myelin-associated proteins (MAG and MBP) in developing mouse brain. ENPP6, MAG and MBP were detected from P4, P8 and P12, respectively. (**D**) Immunohistochemical analysis of ENPP6 in developing mouse brain. ENPP6 expression was evaluated in developing mice brains from P2, P4, P12 and P14. The nucleus of each cell was visualized by hematoxylin staining. Note that ENPP6 is localized around the nucleus at P2 and P4 (arrows). In contrast, ENPP6 is strongly expressed in the myelin sheath but rarely in the cell bodies or perinuclear region at P12 and P14. (**E**) Expression of ENPP6 protein during the differentiation of OPCs to oligodendrocytes. 24, 48, 72 and 96 h after thyroid treatment, ENPP6 expression was examined. (**F**–**H**) Immunofluorescence analysis of ENPP6 during oligodendrocyte differentiation. (**F**) ENPP6 (red) and the OPC-specific marker, NG2 (green). (**G**) ENPP6 (green) and MAG (red). Notably, MAG was not expressed in the cell bodies expressing ENPP6 at 48 h after hormonal treatment (arrows), whereas both ENPP6 and MAG were expressed at 72 h (arrows). (**H**) ENPP6 (green) and the astrocyte marker, GFAP (red). Astrocytes do not express ENPP6. (**I**) Immunohistochemical analysis of ENPP6 in adult mouse liver. A magnified view was also shown. (**J**) Immunofluorescence analysis of ENPP6 (red) and CD146 (green) in the liver.

To understand the physiological roles of ENPP6, we first examined the expression pattern and tissue distribution of ENPP6 in adult mice. ENPP6 was predominantly expressed in kidney and brain and moderately expressed in heart and other tissues (Figs 1A and S1A). In the brain and spinal cord, ENPP6 was mainly expressed in the white matter (Fig. 1B). ENPP6 was not detected at any of tissues of ENPP6 knockout (KO) mice (Fig. S2). The expression pattern was similar to that of myelin basic protein (MBP), a marker of myelin. In oligodendrocytes, ENPP6 was predominantly detected in the myelin sheath but not in the cell bodies (Fig. 1B). ENPP6 was not expressed in neurons and other glial cells (Fig. 1B). Unlike MBP, which was expressed both in Schwann cells and oligodendrocytes, ENPP6 was not detected in Schwann cells in femoral nerves (Fig. 1B). In developing brain, oligodendrocytes start to express ENPP6 prior to MBP and myelin-associated glycoprotein (MAG), another myelin marker (Figs 1C and S1B). The following experiments using immunostaining techniques and highly-purified oligodendrocyte precursor cells (OPCs) revealed that ENPP6 is a novel and early oligodendrocyte differentiation marker. At postnatal day 2 (P2) and P4, ENPP6 was immunohistochemically detected in immature oligodendrocytes, where it was predominantly found both in the processes and cell bodies (Fig. 1D). In contrast, at P12 and P14, ENPP6 was found mainly in myelin sheaths (Fig. 1D). The expression of ENPP6 was not observed in immature OPCs, but it was dramatically increased after the treatment with thyroid hormones (T3 and T4), which induce OPC differentiation into oligodendrocytes (Fig. 1E–G). ENPP6 was not expressed in other nerve cells such as astrocytes (Fig. 1H).

In the kidney, ENPP6 was predominantly expressed in the luminal side of the renal tubule in the cortex (Fig. S3). Staining of serial sections using a tubule-specific marker revealed that ENPP6 signals overlapped with those of Aquaporin-1, a proximal renal tubule marker, indicating that ENPP6 is expressed in the proximal renal tubules in the kidney.

Although the expression level of ENPP6 was low in the liver as judged by western blot analysis (Fig. 1A), immunohistochemical analysis showed that ENPP6 was specifically expressed in tubule-like structures in the liver (Fig. 1I). Further, immunostaining with antibody against CD146, a marker of liver sinusoidal endothelial cells, revealed that ENPP6 is expressed in liver sinusoidal endothelial cells (Fig. 1J).

### ENPP6 KO mice developed fatty liver and myelin sheath abnormalities

**Figure 2:**
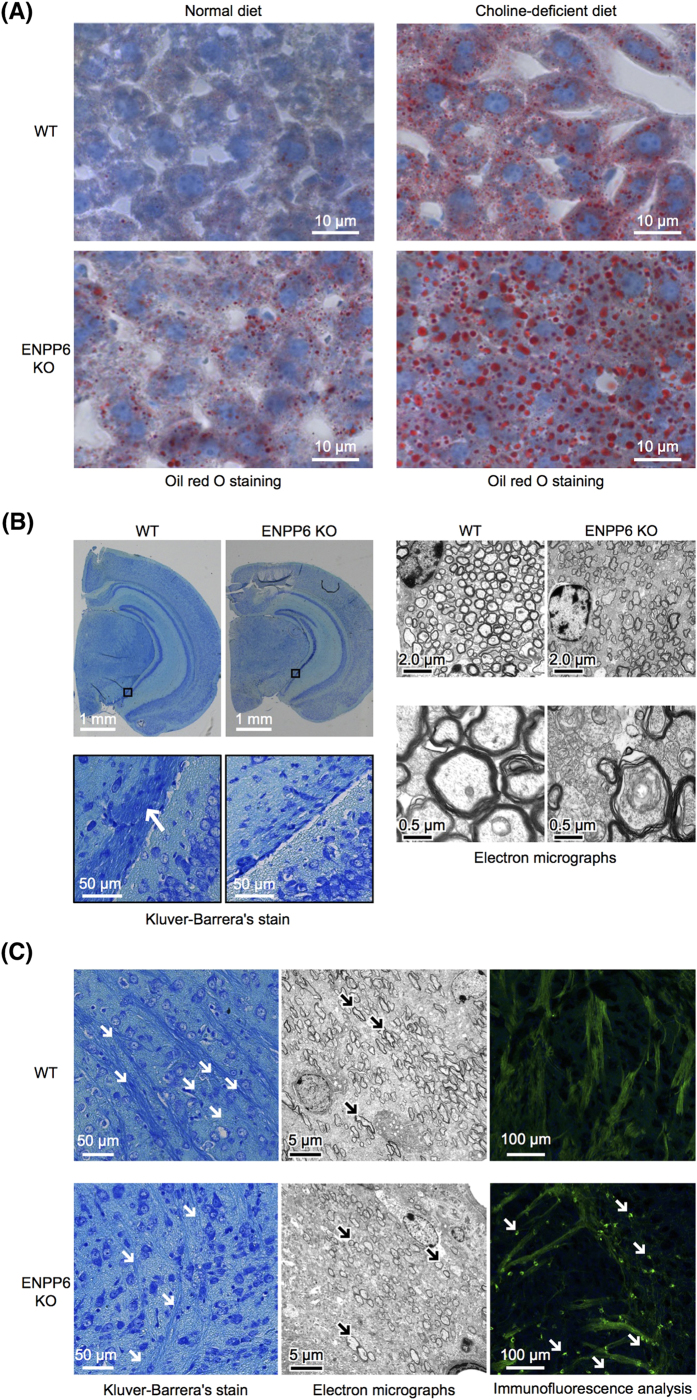
Choline deficiency-like phenotypes of ENPP6 KO mice (**A**) ENPP6 KO mice are prone to develop fatty liver. Liver section from wild-type and ENPP6 KO mice were stained with oil red O. Mice were also fed with choline-deficient diet and the liver section was examined. (**B**,**C**) ENPP6 KO mice show abnormal development of myelin. (**B**) Kluver-Barrera’s staining of brain sections from adult wild-type and ENPP6 KO mice (left four panels). Electron micrographs of myelin (right four panels). Magnified views are shown in the lower panels. Layers of myelin sheath are indicated by arrows. (**C**) Kluver-Barrera’s staining of brain sections from P14 wild-type and ENPP6 KO mice (left panels). Electron micrographs of myelin (center panels). Immunofluorescence analysis of MAG in myelin (right panels). Note that myelin fibers and sheath are less developed and that immature oligodendrocytes with round shapes are evident in ENPP6 KO mice.

To explore the pathophysiological roles of ENPP6, we established ENPP6 KO mice (Fig. S4). ENPP6 KO mice gave birth according to the Mendelian rule and were apparently normal. However, oil red O staining, which detects deposition of neutral lipids, revealed that ENPP6 KO mice exhibit mild symptoms of fatty liver, the most known symptoms induced by choline-deficiency (Fig. 2A). Consistently, ENPP6 KO mice were more susceptible to a choline-deficient diet and exhibited severe fatty liver phenotypes at the early stage (Fig. 2A), suggesting that ENPP6 is involved in choline metabolism in the liver.

We next examined the structure of myelin in ENPP6 KO mice, as ENPP6 was highly expressed in myelin. The Kluver-Barrera staining indicated that the myelin sheath was less developed in ENPP6 KO mice than in wild-type mice (Fig. 2B). Further, electron microscopic images revealed a decrease in the number of myelin sheath layers in ENPP6 KO mice. A similar decrease of myelin sheath was observed in developing myelin at P14 as judged by electron micrography (Fig. 2C). At P14, in which the formation of myelin sheath is most active and the expression of ENPP6 reaches the plateau, many MAG-positive oligodendrocytes remained round, indicating that they failed to wrap axon to form myelin sheath (Fig. 2C). Notably, myelin is rich in lipids, especially choline-containing phospholipids such as SM^24^, suggesting that ENPP6 is involved in choline metabolism in the brain. Together, our analyses of ENPP6 KO mice indicate that ENPP6 plays important roles in choline metabolism in liver and brain.

### ENPP6 hydrolyzes α-GPC and contributes to the choline supply *in vitro*

**Figure 3:**
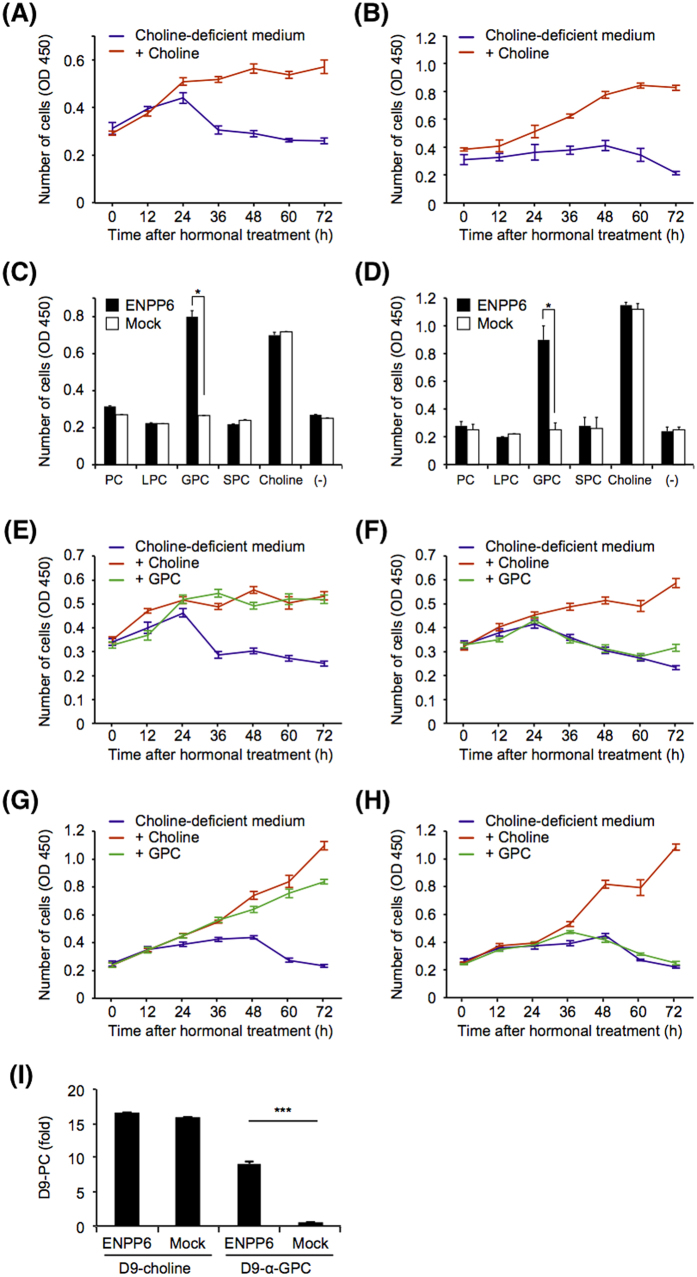
Enforced expression of ENPP6 enables the cell to utilize GPC as a choline source. (**A**,**B**) The proliferation of Neuro2a (**A**) and NIH3T3 (**B**) cells was attenuated in choline-deficient medium. The number of cells was restored by addition of choline into choline-deficient medium. (**C**,**D**) Addition of GPC restored only the proliferation of ENPP6-expressing Neuro2a (**C**) and NIH3T3 (**D**) cells in the choline-deficient medium. ENPP6 was stably expressed in Neuro2a and NIH3T3 cells using a retroviral expression system. Stable transformants (Neuro2a (**C**) and NIH3T3 (**D**)) were cultured in choline-deficient DMEM containing PC, LPC, GPC, SPC or choline (14.5 μM each) as a choline source. The numbers of the cultured were examined at 72 h using the MTT method. Data are shown as mean ± SEM (*p < 0.05, n = 3). (E–H) The numbers of ENPP6 stable transformants (Neuro2a (**E**) and NIH3T3 (**G**)) and their control cells (Neuro2a (**F**) and NIH3T3 (**H**)) cultured with GPC or choline were examined at indicated time. Data are shown as mean ± SEM (n = 4). (I) Incorporation of D-choline into PC fraction. ENPP6 was stably expressed in McA-Rh7777 rat hepatomas using a retroviral expression system. McA-Rh7777 cells stably carrying ENPP6 or control retroviral genes were cultured in choline-deficient medium both in the presence or absence of D-choline or D-α-GPC. After 24 h of culture, D-PC in the cells was extracted and analyzed using LC-MS/MS. Data are shown as mean+SEM (***p < 0.001, n=4).

To understand the molecular mechanism by which ENPP6 participates in choline metabolism at the cellular level, we examined the effects of the overexpression of ENPP6 (Fig. S5) and addition of choline-containing compounds on the proliferation of cultured cells in choline-deficient medium. The proliferation of both Neuro2a and NIH3T3 cells was severely reduced in choline-deficient medium, and the number of the cells dramatically decreased 72 h after the choline deprivation (Fig. 3A,B). These results are not surprising in view of the fact that choline is an essential factor for cultured cells. Addition of choline to the choline-deficient medium restored the proliferation of both cultured cells (Fig. 3A–D). Addition of other choline-containing compounds such as PC, LPC, SPC and α-GPC failed to restore the proliferation (Fig. 3C,D). Similarly, the proliferation of Neuro2a and NIH3T3 cells that stably express ENPP6 was inhibited in the choline-deficient medium, and addition of PC, LPC and SPC to the medium was ineffective (Fig. 3C,D). In contrast, addition of α-GPC restored the proliferation of ENPP6-expressing cells in the choline-deficient medium (Fig. 3C–H). These results indicate that ENPP6-expressing cells could use α-GPC, but not other choline-containing compounds, as a choline source.

We thus examined the metabolism of α-GPC in ENPP6-expressing cells, using deuterium-labeled α-GPC (D-α-GPC), which is chemically-synthesized from deuterium-labeled choline (D-choline). In this experiment, we used McA-Rh7777 rat hepatoma cells as a model of hepatocytes. When D-choline was added to the culture medium, D-choline was incorporated into the PC fraction similarly in both ENPP6-expressing and control cells (Fig. 3I). In contrast, when D-α-GPC was added to the culture medium, PC-containing D-choline was detected almost exclusively in ENPP6-expressing cells (Fig. 3I), indicating that D-choline was incorporated into ENPP6-expressing but not control cells. These results suggested that at the cellular level, ENPP6 hydrolyzes α-GPC to produce phosphocholine as a choline source, thereby contributing to the choline needed by the liver and needed for myelin synthesis.

### Accumulation of β-GPC in the urine of ENPP6 KO mice

**Figure 4:**
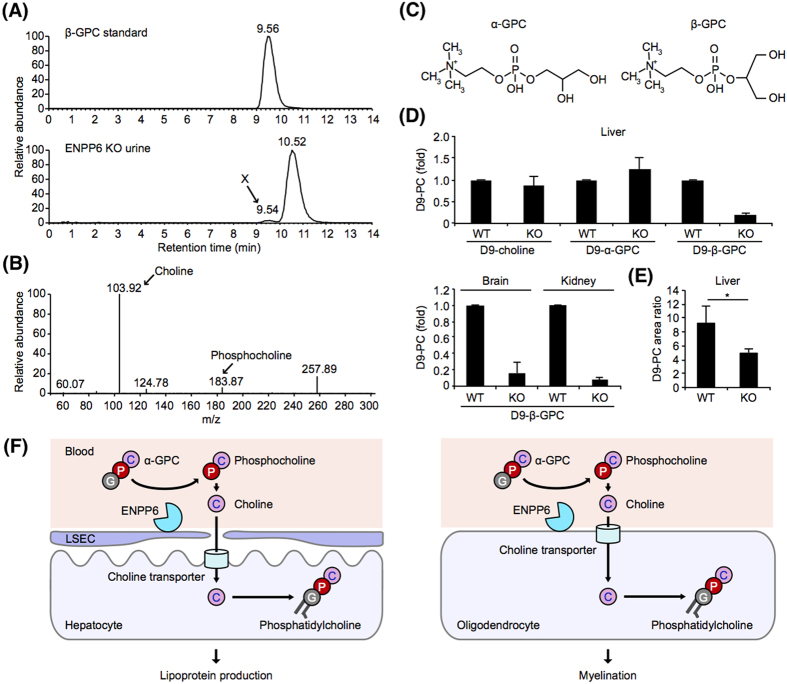
Identification of β-GPC and its metabolism in ENPP6 KO mice. (**A**) Identification of β-GPC in the urine of ENPP6 KO mice. Mass chromatogram of chemically synthesized β-GPC (monitored ion *m/z* 257.9) on normal-phase column chromatography (upper). Mass chromatograms of α-GPC (retention time 10.52 min) and compound X (retention time 9.54 min) detected in urine of ENPP6 KO mice (monitored ion *m/z* 257.9) showed that compound X has an identical retention times with β-GPC (lower). (**B**) Mass spectrum of compound X, showing compound X has choline and phosphocholine residues in its structure. (**C**) Structures of α- and β-GPC. (**D**,**E**) Incorporation of D-choline, D-α-GPC and D-β-GPC to phosphatidylcholine in various tissues. D-choline, D-α-GPC or D-β-GPC was injected into wild-type and ENPP6 KO mice via *i.v.* (**D**) or *i.p.* (**E**). After 3 h, phospholipids in each tissue were extracted and analyzed by LC-MS/MS. Data are shown as mean+SD (* *p* < 0.05, n=5) (**F**) Schematic model for ENPP6-mediated uptake of GPC in hepatocyte (left) and oligodendrocyte (right).

We next examined GPC metabolism in ENPP6 KO mice. LC-MS/MS analysis revealed that the levels of endogenous choline-containing compounds such as free choline, betaine, α-GPC, LPC and PC were almost similar in the plasma and at the tissues of wild-type and ENPP6 KO mice (Fig. S6A–C). However, we found that the urine level of a choline-containing compound with an *m/z* value identical to that of α-GPC was higher in ENPP6 KO mice than in wild-type mice (Fig. S7). The choline-containing compound migrated slightly faster on the LC column than α-GPC (Fig. S7). LC-MS/MS analysis identified this as β-GPC, which accounted for a minor contribution (~1%) to total GPC (Fig. 4A–C). Kinetic analyses using recombinant ENPP6 protein indicated the enzyme showed similar affinities to both GPCs (Fig. S8A). In addition, when added to the culture media of ENPP6-expressing rat hepatoma cells, both D-α-GPC and D-β-GPC were similarly degraded to D-choline and converted to D-PC (Fig. S8B,C). Unlike D-α-GPC, D-β-GPC was found to be extremely stable in mouse plasma. D-α-GPC was degraded in mouse blood just as fast as it was in the blood from ENPP6 KO mice (Fig. S9A). D-α-GPC was quickly converted to D-choline (Fig. S9A), suggesting that α-GPC, but not β-GPC, is hydrolyzed by a phosphodiesterase other than ENPP6 in the blood.

### Both α- and β-GPC are hydrolyzed by ENPP6 and utilized as choline sources *in vivo*

When D-α-GPC and D-β-GPC were injected *i.v.* into wild-type mice, D-PC was first detected in the liver and then in plasma (data not shown). These results indicated that both GPCs are incorporated into liver and converted to PC, which is then secreted into plasma as lipoproteins. When D-α-GPC or D-choline was injected *i.v.*, the similar levels of D-PC were detected in the liver of wild-type and ENPP6 KO mice (Fig. 4D), consistent with the observation that α-GPC is hydrolyzed similarly in the plasma of both mice by an unknown mechanism. In contrast, when D-β-GPC was injected, the D-PC levels in the liver, brain and kidney were significantly lower in ENPP6 KO mice than in wild-type mice (Fig. 4D). α-GPC that was injected *i.p.* was maintained at a higher concentration in plasma than α-GPC injected *i.v.* (Fig. S9B). Only when D-α-GPC was injected *i.p.* was significantly less converted to D-PC in the ENPP6 KO mice (Fig. 4E), clearly indicating that ENPP6 is involved in α-GPC metabolism. Together, the *in vivo* and *in vitro* evidence suggest that ENPP6 on the cell surface hydrolyzes GPC to phosphocholine and contributes to the choline supply (Fig. 4F).

### Crystal structure of ENPP6

**Figure 5:**
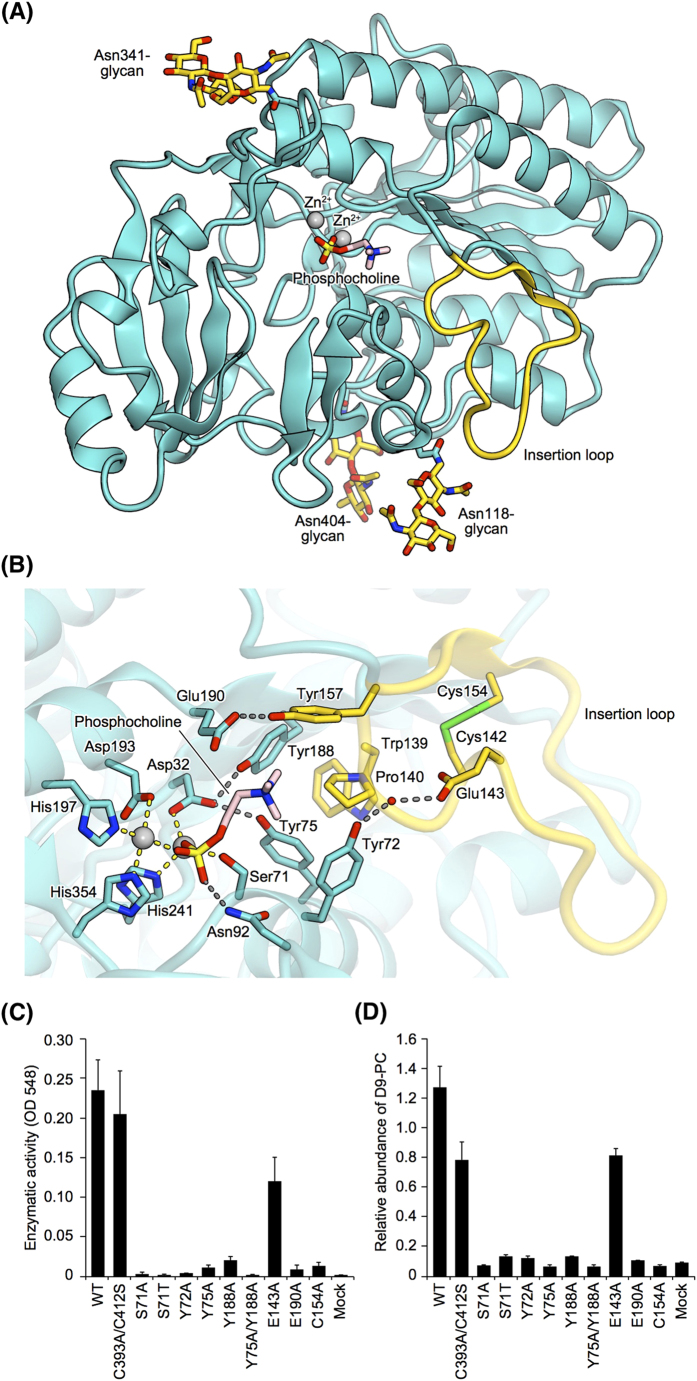
Crystal structure of ENPP6. (**A**) Crystal structure of the PDE domain of ENPP6 in complex with phosphocholine. *N*-linked glycans and the phosphocholine are shown as gold and light pink sticks, respectively. (**B**) Active site of ENPP6 in complex with phosphocholine. Hydrogen bonds and coordinate bonds are shown as dashed gray and yellow lines, respectively. A water molecule is shown as a red sphere. The insertion loop is shown in gold and the bound zinc ions are shown as gray spheres in (**A**,**B**). (**C**) α-GPC-hydrolyzing activities of the ENPP6 mutants. (**D**) Choline uptake in cultured cell lines that express wild-type or mutant ENPP6 proteins. Data are shown as mean ± SEM (n = 3).

To elucidate the choline recognition mechanism of ENPP6, we attempted to solve the crystal structures of ENPP6. The extracellular PDE domain of mouse ENPP6 (residues 24–415) exists as a mixture of a monomer, dimer and higher-order oligomer, whereas the C393A/C412S mutant predominantly exists as a monomer^25^. The C393A/C412S mutant showed α-GPC-hydrolyzing activity comparable to the activities of the monomer and dimer of wild-type ENPP6 (Fig. 5C), indicating that neither the oligomeric state nor the C393A/C412S mutation affects the enzymatic activity. We thus determined the crystal structures of the C393A/C412S mutant in the apo form (without substrates) at 2.0 Å resolution (Fig. S10A). Previous crystal structures of ENPP1, ENPP2 and ENPP4 in complex with their reaction products (AMP, LPA and AMP, respectively) provided structural insights into their substrate recognition mechanisms^19^,^20^,^21^,^26^,^27^ (Fig. S11). Accordingly, we also determined the crystal structure of ENPP6 in complex with phosphocholine, a reaction product, at 1.8 Å resolution (Fig. 5A). Since the two structures are virtually identical (root-mean-square deviation [rmsd] of 0.23 Å for aligned Cα atoms), we will hereafter describe the ENPP6–phosphocholine complex structure, unless otherwise stated.

The structure of the PDE domain of ENPP6 is similar to the structures of the PDE domains of ENPP1 (PDB code 4GTW, 32% identity, rmsd of 1.6 Å for aligned 364 Cα atoms)^19^, ENPP2 (PDB code 3NKM, 28% identity, rmsd of 1.7 Å for aligned 340 Cα atoms)^20^, and ENPP4 (PDB code 4LR2, 33% identity, rmsd of 1.4 Å for aligned 359 Cα atoms)^21^ (Fig. S11). Although electron densities of four glycans linked to Asn100, Asn118, Asn341 and Asn404 are observed in the apo form, the electron density of the Asn100-linked glycan was not observed in the complex, probably due to the flexibility of the Asn100-linked glycan (Fig. S10A). As observed in the structures of ENPP1, ENPP2 and ENPP4, two zinc ions are coordinated by conserved active-site residues (Fig. 5B). One zinc ion is coordinated by Asp193, His197 and His354, while the other is coordinated by Asp32, His241 and the catalytic nucleophile Ser71 (Fig. 5B).

### Choline recognition mechanism

An electron density corresponding to the bound phosphocholine was observed in the vicinity of the zinc ions in the catalytic pocket (Fig. S10B). The choline moiety of the phosphocholine is accommodated in a choline-binding pocket formed by the side chains of Tyr72, Tyr75, Tyr157 and Tyr188, while the phosphate group of the phosphocholine is located in the vicinity of the two zinc ions (Figs 5B and S10B). Tyr72, Tyr75 and Tyr188 provide π-cation interactions with the positively-charged choline moiety. Furthermore, Asp32 and Glu190 electrostatically interact with the choline moiety. Tyr72 forms a water-mediated hydrogen bond with Glu143, while Tyr75/Tyr188 and Tyr157 form direct hydrogen bonds with Asp32 and Glu190, respectively, thereby stabilizing the conformation of the choline-binding pocket. In addition, Trp139 and Pro140 in the “insertion loop” (residues 139–157) interact with the side chains of Tyr72, Tyr75, Tyr157 and Tyr188. The conformation of the “insertion loop” is stabilized by a disulfide linkage between Cys142 and Cys154.

To verify the functional significance of the choline-binding pocket, we introduced mutations to the C393A/C412S mutant, purified the mutants, and measured their α-GPC-hydrolyzing activities. Among the mutants tested, the Y157A mutant was not expressed in the culture supernatants of HEK293T cells, which suggests that Tyr157 is essential for correct protein folding. The other mutants eluted as a single peak from the gel-filtration column, confirming their structural integrity (data not shown). The Y72A, Y75A, Y188A and E190A mutants exhibited the remarkably reduced α-GPC-hydrolyzing activities, as compared with the wild-type and C393A/C412S mutant proteins (Fig. 5C), confirming the importance of Tyr72, Tyr75, Tyr188 and Glu190 for choline recognition. The activities of the C154A mutants were reduced, indicating the importance of the disulfide linkage between Cys142 and Cys154 for the choline-binding pocket formation. The Tyr72, Tyr75, Cys142 and Cys154 are evolutionarily conserved among ENPP6 proteins, but not among other ENPP family members (Fig. S12A), highlighting the importance of the tyrosine residues and the disulfide linkage in the “insertion loop” for choline recognition. The catalytic nucleophile is a serine residue in ENPP6 (Ser71 in mouse ENPP6), whereas it is a threonine residue in other ENPP family members (Figs S12B and S13A–D). Indeed, the S71A and S71T mutants of ENPP6 showed almost no α-GPC-hydrolyzing activities (Fig. 5C). In the crystal structure, the side-chain Cβ atom of Ser71 forms van der Waals interactions with the side chain of Tyr75, and contributes to the choline-binding pocket formation (Fig. S13D), suggesting that the side-chain methyl group of a threonine residue at this position would generate steric clashes with Tyr75. These observations provided a structural explanation for why only ENPP6 has a serine residue as a catalytic nucleophile among the ENPP family members.

To examine the physiological significance of choline recognition by ENPP6 for the choline metabolism, we evaluated choline uptake in cultured cells that express wild-type or mutant ENPP6 proteins. Wild-type ENPP6 expressed in cultured hepatomas hydrolyzed both α- and β-GPC for the biosynthesis of PC (Fig. S8B,C). In contrast, the mutant ENPP6 proteins, which had impaired α-GPC-hydrolyzing activities (Fig. 5C), failed to support such choline metabolism (Fig. 5D). Together, our structural and mutational analyses reveal that specific recognition of the choline moiety of GPC is required for ENPP6-mediated choline metabolism.

## Discussion

GPC is present in various biological fluids such as blood and cerebrospinal fluids at the several μM level, but its *in vivo* roles remain unclear. Here, we showed that GPC is hydrolyzed to phosphocholine by ENPP6, which is present on the extracellular surface of certain cells such as oligodendrocytes and liver sinusoid endothelial cells. We confirmed that the phosphocholine was quickly converted to free choline in various cell types (data not shown), possibly by non-specific phosphatases, indicating that it is used as a choline source for synthesis of choline-containing phospholipids such as PC. In addition, we showed that ENPP6-deficient mice have impaired myelin structure and are prone to having a fatty liver, which are typical symptoms of choline deficiency. Together, these results strongly suggest that ENPP6 serves as a choline-supplying enzyme.

ENPP6 was found to be an early marker of oligodendrocyte differentiation. In developing brain, ENPP6 started to express prior to MAG and MBP (Fig. 1C), later oligodendrocyte differentiation markers. In cultured OPCs, ENPP6 started to express when they were stimulated to differentiate to oligodendrocytes (Fig. 1E,F). In the developing brain, oligodendrocytes require large quantities of choline to make lipid-rich myelin. The present data strongly suggest that ENPP6 contributes to supplying choline for oligodendrocytes (Fig. 4F). This idea is supported by the observations that the expression pattern of ENPP6 in the developing brain was very similar to the expression pattern of several choline-related molecules (CTP-phosphocholine transferase (CTα), high-affinity choline transporter (CHT1), Cyclic-nucleotide phosphodiesterase (CNPase), low-affinity choline transporter (CTL1α and CTL1β) and sphingomyelin synthases (SMS1 and SMS2)) (Fig. S14). At the initial stage of differentiation, OPCs are surrounded by neurons that express high-affinity choline transporters at high levels, which may make it difficult for OPCs to incorporate free choline. Thus, it is reasonable to assume that OPCs, unlike ENPP6-negative neurons, have an alternative pathway to acquire choline.

In the liver, ENPP6 is expressed in sinusoidal endothelial cells, which mediate the transfer of substrates between the sinusoidal blood and hepatocytes, and thus are important for hepatic function. Like the brain, the liver requires large quantities of choline. As liver cells continuously synthesize and secrete PC as a form of lipoprotein (Fig. 4F), ENPP6 appears to have a role in supplying choline in the liver. Similar ENPP6-mediated choline uptake was observed in the kidney (Fig. 4D). ENPP6 was expressed in epithelial cells forming the lumen of the proximal tubules, which is where choline in the primary urine is reabsorbed. Thus, it is possible that ENPP6 in the kidney contributes to the reabsorption of choline by hydrolyzing GPC in the primary urine. The present data suggest that cells that require choline uptake for growth, for phospholipid synthesis or for re-use, have another pathway to acquire choline that is mediated by ENPP6. In other words, many organisms may have evolved multiple pathways for acquisition of choline, which is requisite as a vitamin-like nutrient. We detected a structural isomer of α-GPC, viz. β-GPC, in the urine of ENPP6 KO mice. We postulate that PC is converted to α-GPC by sequential degradation by phospholipases. The detection of β-GPC indicates the presence of β-PC *in vivo*. Until now, it has been thought that β-PC could be produced only in the laboratory. We also found that β-GPC, unlike α-GPC, is unexpectedly and highly stable in the circulation, although both GPCs showed similar biochemical properties as substrates for ENPP6. This explains why only β-GPC was detected in the urine of ENPP6 KO mice and was effectively incorporated in an ENPP6-dependent manner.

The crystal structure of the ENPP6–phosphocholine complex showed that ENPP6 recognizes the choline moiety of phosphocholine, which strongly supports the idea that ENPP6 recognizes the choline moiety of its substrates, such as GPC and LPC, and hydrolyzes them to produce phosphocholine in a similar manner. In the crystal structure, the choline moiety is recognized by four conserved tyrosine residues and one conserved glutamic acid residue (Fig. 5B). Our biochemical and biological analyses confirmed the functional significance of the choline-binding pocket for choline recognition (Fig. 5C,D). The aromatic and acidic residues forming the choline-binding pockets are conserved completely in ENPP6 proteins from various species (Fig. S12A) and are also conserved in other choline-binding proteins^28^ (Fig. S15). In the crystal structures of ENPP6 and other choline-binding proteins, the choline moiety of ligands form π–cation interactions with surrounding multiple aromatic residues, with an acidic residue serving as a counterion to the positively charged choline moiety. Thus, the present structure of ENPP6 provides a new example of the choline recognition mechanism of choline-binding proteins, and reinforces the notion that protein of diverse sequences have evolved convergently to recognize the choline moiety of their substrates, using choline-binding pockets of a similar structural feature.

**Figure 6:**
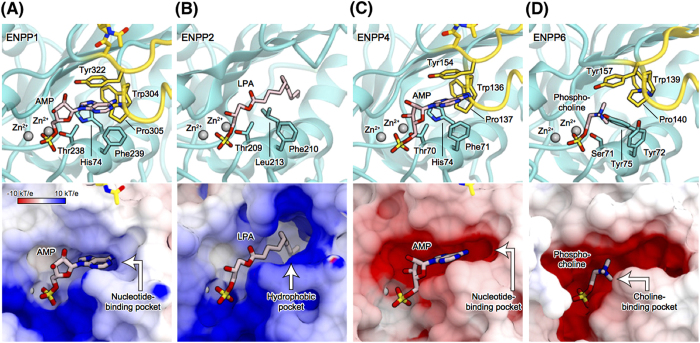
Active sites of the ENPP family members. (**A**–**D**) Active sites of ENPP1 in complex with AMP (PDB ID 4GTW) (**A**), ENPP2 in complex with 14:0-LPA (PDB ID 3NKN) (**B**), ENPP4 in complex with AMP (PDB ID 4LQY) (**C**), and ENPP6 in complex with phosphocholine (**D**). The structures are shown in ribbon representations (top panels) and in electrostatic surface potentials (contoured from −10 kT/e [red] to +10 kT/e [blue]) (bottom panels). The bound products are shown as light pink sticks. The insertion loops are shown in gold in (**A**,**C**,**D**).

Previously determined crystal structures of ENPP1, ENPP2 and ENPP4 provided insights into their substrate recognition mechanisms^19^,^20^,^21^,^26^,^27^. A structural comparison of ENPP6 and other ENPPs offers an explanation of why among the family members only ENPP6 specifically recognizes the choline moiety of substrates. ENPP1 and ENPP4 hydrolyze nucleotide substrates to produce AMP and pyrophosphate^16^,^29^. In the crystal structures of mouse ENPP1^19^, the adenine base of AMP is recognized by the conserved phenylalanine and tyrosine residues (Phe239 and Try322) through stacking interactions (Fig. 6A). Similarly, in the crystal structure of mouse ENPP4^21^, the adenine base is recognized by the conserved phenylalanine residues (Phe71 and Try154) through stacking interactions (Fig. 6C). ENPP6 Tyr72/Tyr157 correspond to ENPP1 Phe239/Tyr322 and ENPP4 Phe71/Tyr154, respectively (Fig. 6D). These observations suggested that ENPP6 cannot hydrolyze nucleotide substrates, due to steric clashes between the hydroxyl group of Tyr72 and the base moiety of nucleotide substrates.

ENPP2 hydrolyzes LPC into LPA and choline by its lysoPLD activity^12^, whereas ENPP6 hydrolyzes LPC into monoacylglycerol and phosphocholine by its lysoPLC activity. Unlike the other ENPP family members, ENPP2 lacks the “insertion loop” and instead has a hydrophobic pocket, which accommodates the acyl chain of LPC substrates^20^ (Fig. 6B). In contrast, ENPP6 has the “insertion loop”, which occludes a hydrophobic pocket and contributes to the formation of the negatively charged choline-binding pocket, which accommodates the positively charged choline moiety of LPC (Fig. 6D). These observations indicated that LPC binds to the catalytic pockets of ENPP2 and ENPP6 in the reverse orientation, thereby explaining their distinct activities for the LPC substrates.

## Experimental procedures

### Materials

LPC and phosphatidylcholine (PC) extracted from chicken eggs were purchased from Avanti Polar Lipids (Alabaster, AL). Sphingosylphosphorylcholine (SPC) was from Biomol Research Laboratories (Plymouth Meeting, PA). Glycerophosphorylcholine (cadmium free) was from Kanto Kagaku (Tokyo, Japan). Other chemicals were from Wako Pure Chemical Industries, Ltd. (Osaka, Japan).

### Generation of ENPP6 knockout mice

ENPP6 KO mice used in this study were established by replacing exon1 containing the initiation methionine with a Neo-cassette, described in the supportive information. We established two mouse lines from independent ES clones. Because two lines showed similar phenotypes, we mainly used one line. All experiments using mice were carried out based on the Tohoku University guidelines for animal experiments, after permission was granted by the Committee of Animal Use and Care of the Tohoku University.

### Monoclonal antibody to mouse ENPP6

The rat monoclonal antibody against mouse ENPP6 used in this study (clone 10B10) was purified from 10B10-producing rat-mouse hybridoma cells (supportive information).

### Western blotting

Protein samples were subjected to SDS-PAGE and transferred to nitrocellulose membranes (Bio-Rad, Hercules, CA). After blocking with skim milk, the membrane was incubated with anti-mouse ENPP6 monoclonal antibody (10B10), rabbit anti-MAG polyclonal antibody or rabbit anti-Myelin basic protein (MBP), and then treated with the corresponding second antibodies conjugated with horseradish peroxidase (American Qualex, Inc, San Clemente, CA). Proteins bound to the antibodies were visualized with an enhanced chemiluminescence (ECL) kit (Amersham Biosciences, Piscataway, NJ).

### Immunohistochemistry

Mouse tissues were dehydrated and embedded in paraffin using standard techniques. Five-micrometer sections were used in all histological studies. Immunohistochemistry was performed using an avidin/biotin blocking kit (Vector, Burlingame, CA) and a VECTASTAIN ELITE ABC kit (Vector), according to the manufacturer’s instructions. Nonspecific immunoglobulin binding was blocked with phosphate-buffered saline containing 10% normal rabbit or goat serum. The sections were incubated overnight at 4 °C with first antibodies (rat anti-mouse ENPP6 monoclonal antibody (10B10) or rabbit anti-myelin basic protein (MBP)), placed on glass slides, washed, incubated for 1 h at room temperature with 1 to 1,000 dilution of biotin rabbit anti-rat IgG or goat anti-rabbit IgG, treated with avidin-biotin-peroxidase complex (ABC ELITE), washed, developed with dimethylaminoazobenzene, and counterstained with hematoxylin.

### Quantitative Real-time RT-PCR

Total RNA from cells was extracted using ISOGEN (Nippongene, Toyama, Japan) and reverse-transcribed using the SuperScript first-strand synthesis system (Invitrogen). PCR reactions were performed using an ABI Prism 7000 sequence detection system (Applied Biosystems). The transcript number of mouse GAPDH was quantified, and each sample was normalized on the basis of GAPDH content. Oligonucleotide primers for PCR were listed in Table S1.

### Oligodendrocyte progenitor cell (OPC) culture

Mouse oligodendrocyte progenitor cells (OPCs) cultures were established by a modification of the methods of Yang^30^ and Seiwa^31^ (see supportive information).

### Immunofluorescence experiment

OPCs were fixed with ice-cold methanol, incubated with first antibodies (anti-mouse ENPP6 (rat monoclonal antibody, 10B10), anti-myelin associated glycoprotein (MAG) (rabbit polyclonal antibody), anti-NG2 (rabbit polyclonal antibody (CHEMICON International, AB5320)) or anti-glial fibrillary acidic protein (GFAP) (rabbit polyclonal antibody (CHEMICON International., AB5804), and incubated with Alexa Fluor 488-goat anti-rat IgG (Molecular Probes, Inc., Eugene, OR) or Alexa Fluor 594-anti-rabbit IgG (Molecular Probes, Inc.). Bound antibodies were detected with a fluorescence microscope (DMIRE2, Leica Microsystems AG, Wetzlar, Germany).

### Development of fatty liver

Mice were fed with normal or choline-deficient diet (CD diet, Dyets, Bethlehem, PA, U.S.A.) for one week. For evaluation of fatty liver, liver tissue section (10 μm) was stained with oil red O and hematoxylin.

### Electron microscopy

Brain tissues from P14 and adult mice were isolated and fixed in 4% PFA. For electron microscopy, the brain tissues were postfixed in 1% OsO_4_ and 0.8% K_4_Fe(CN)_6_ in 0.1 M sodium cacodylate buffer and embedded in Epon 812 (TAAB Laboratories Equipment Ltd., Berkshire, England). Ultrathin sections (85 nm) were examined with an H-7100 system (Hitachi Hi-Technologies Co., Tokyo, Japan).

### Quantification of choline-containing compounds and phospholipids by LC-MS/MS

Choline-containing compounds and phospholipids were quantified by LC-MS/MS as previously described^32^ with some modifications (see supportive information).

### Choline incorporation assay

ENPP6-expressing cells were generated with a retroviral system (see supportive information). Choline incorporation activity was measured using α-GPC in ENPP6-expressing cells in DMEM supplemented with 1% lipoprotein-depleted serum (LPDS). LPDS was prepared from FBS as described previously^33^. It is noted that the obtained LPDS does not contain free choline or GPC. Cells were incubated with 10 μM D-α-GPC for 24 h and then lipids were extracted by methanol. The level of PC was determined by LC-MS/MS.

### Cell proliferation assay

Neuro2A and NIH3T3 cells were seeded at a density of 2,000 cells per well in 24-well plates in DMEM with 10% FBS. After overnight incubation, the medium was changed to choline-free DMEM (CSTI, Japan) supplemented with 1% LPDS. Cells were treated with each choline-containing compound (14.5 μM) and cultured for several days. Cell proliferation was assessed by Cell Counting Kit-8 (Dojindo, Kumamoto, Japan).

### α-GPC-hydrolyzing assay for the ENPP6 mutants

Site-directed mutagenesis was performed using PrimeSTAR Max DNA Polymerase (TaKaRa). The mutants of ENPP6 were expressed as secreted forms in transiently transfected HEK293T cells, and then purified as previously described^25^. Each ENPP6 protein was mixed with α-GPC and calf intestine alkaline phosphatase (20 U/ml) at 37 °C. After 1 h, choline produced from α-GPC hydrolysis was detected enzymatically^12^.

### Chemical synthesis of α-GPC and β-GPC

α-GPC and β-GPC were synthesized as described in supportive information.

### Crystallography

The extracellular PDE domain of mouse ENPP6 C393A/C412S mutant (residues 1–421), which lacks the C-terminal GPI-attachment signal sequence, was expressed and purified as described previously^25^. Crystallization was performed at 20 °C by the vapor diffusion methods. Although the protein was crystallized in the presence of the inhibitor T11^14^, no electron density was observed for the inhibitor. We thus refer to this structure as an apo form. Crystals of the apo form were obtained by mixing 0.1 μl of protein solution (2 mg/ml ENPP6, 2 mM T11, 20 mM Tris-HCl pH 7.5, 150 mM NaCl) and 0.1 μl of reservoir solution (0.2 M NH_4_Cl, 0.1 M sodium acetate pH 5.0, 20% PEG 6000). Crystals were cryoprotected in reservoir solution supplemented with 25% (v/v) ethylene glycol and were flash-cooled in liquid nitrogen. X-ray diffraction data were collected at 100 K on beamline BL41XU at the SPring-8 (Hyogo, Japan) and processed using XDS^34^. The structure was determined by molecular replacement with MOLREP^25^. The search model was built by the Phyre 2 server^18^, using the PDE domain of mouse ENPP1 (PDB ID 4GTW^19^) as a template. Model building and refinement were performed using COOT^35^ and PHENIX^36^. Crystals of ENPP6 in complex with phosphocholine were obtained as described previously^25^. X-ray diffraction data were collected at 100 K on beamline PXI at the Swiss Light Source. The crystal structure of ENPP6 in complex with phosphocholine was determined by molecular replacement, using the apo form of ENPP6 as a search model. Data processing, model building and refinement were performed as described above. Data collection and refinement statistics are provided in Table 1.

## Additional Information

**Accession Numbers**: The atomic coordinates and the structure factors of ENPP6 have been deposited in the PDB ID codes 5EGE (apo) and 5EGH (phosphocholine complex). 

**How to cite this article**: Morita, J. *et al.* Structure and biological function of ENPP6, a choline-specific glycerophosphodiester-phosphodiesterase. *Sci. Rep.* **6**, 20995; doi: 10.1038/srep20995 (2016).

## Supplementary Material

Supplementary Information

## Figures and Tables

**Table 1:** Data collection and refinement statistics.

	Apo ENPP6	ENPP6–Phosphocholine
**Data collection**		
Beamline	SPring-8 BL41XU	SLS PXI
Wavelength (Å)	1.282	1.278
Space group	*P*1	*P*1
Cell dimensions		
*a*, *b*, *c* (Å)	64.3, 78.2, 103.6	63.7, 68.8, 69.7
α, β, γ (°)	90.0, 90.0, 114.6	60.6, 87.0, 68.1
Resolution (Å)	50.0–1.99 (2.02–1.99)	50.0–1.80 (1.91–1.80)
*R*_sym_	0.188 (0.835)	0.058 (0.624)
*I*/σ*I*	6.11 (1.40)	12.8 (1.78)
Completeness (%)	96.6 (93.7)	93.6 (88.5)
Redundancy	3.96 (3.99)	3.54 (3.28)
CC(1/2)	0.99 (0.62)	0.99 (0.75)
**Refinement**		
Resolutions (Å)	38.9–2.00 (2.01–2.00)	45.6–1.80 (1.87–1.80)
No. Reflections	120,105	81,806
*R*_work_/*R*_free_	0.167/0.206	0.175/0.209
No. atoms		
Protein	12,807	6,439
Ligand/ion	394	220
Water	2,048	528
*B*-factors (Å^2^)		
Protein	20.4	31.7
Ligand/ion	38.5	49.9
Water	31.1	38.2
R.m.s. deviations		
Bond length (Å)	0.007	0.007
Bond angles (°)	0.890	0.906
Ramachandran plot		
Allowed region	3.6%	3.9%
Outlier region	0.2%	0.1%

*Values in parentheses are for the highest-resolution shell.
