# Supplementary material for: Structure and biological function of ENPP6, a choline-specific glycerophosphodiester-phosphodiesterase
Source: Sci Rep. 2016 Feb 18;6:20995. doi: 10.1038/srep20995 (PMC4757880; doi:10.1038/srep20995)
Supplement: Supplementary Information [file srep20995-s1.pdf]

Scientific Reports

Supplemental Information

Structure and biological function of ENPP6, a choline-specific glycerophosphodiester-phosphodiesterase

Junko Morita, Kuniyuki Kano, Kazuki Kato, Hiroyuki Takita, Hideki Sakagami, Yasuo Yamamoto, Emiko Mihara, Hirofumi Ueda, Takanao Sato, Hidetoshi Tokuyama, Hiroyuki Arai, Hiroaki Asou, Junichi Takagi, Ryuichiro Ishitani, Hiroshi Nishimasu, Osamu Nureki and Junken Aoki

SUPPLEMENTAL FIGURES

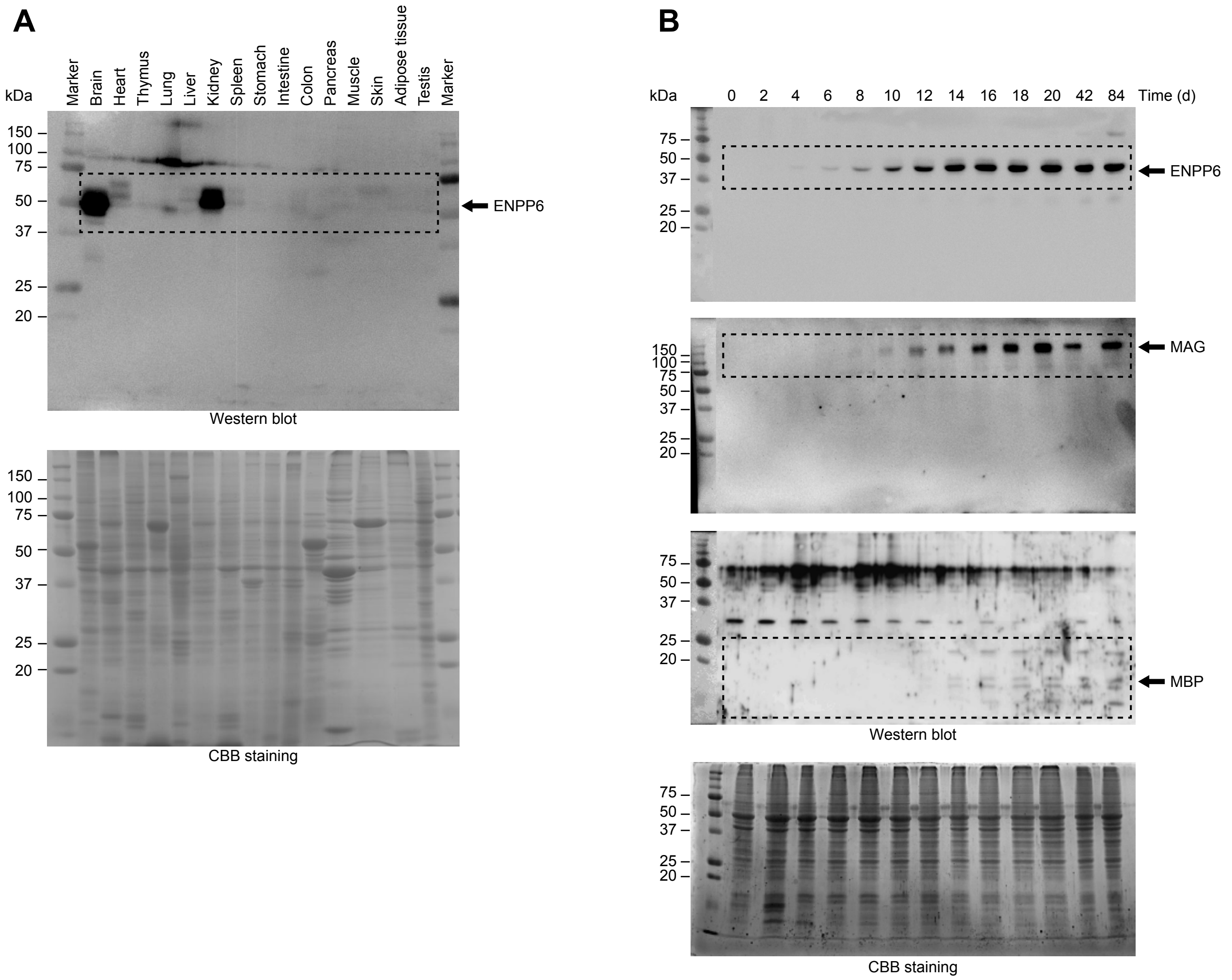

Supplementary Figure 1

Supplementary Figure 1. Full-length blots and gels

(A) Full-length blots and CBB-stained gels for Figure 1A. (B) Full-length blots and CBB-stained gels for Figure 1C. The bands of interest are displayed within the dashed boxes.

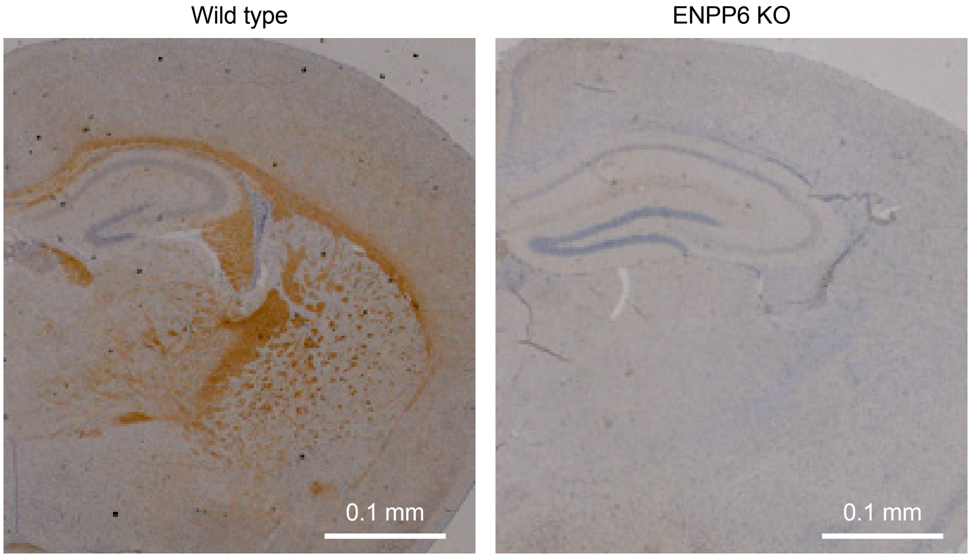

Supplementary Figure 2

Supplementary Figure 2. Specificity of anti-ENPP6 monoclonal antibody.

Brain from wild-type mice and ENPP6 KO mice was fixed and immunostained with anti-ENPP6 antibody. No specific signals were detected in the brain of ENPP6 KO mice.

ENPP6

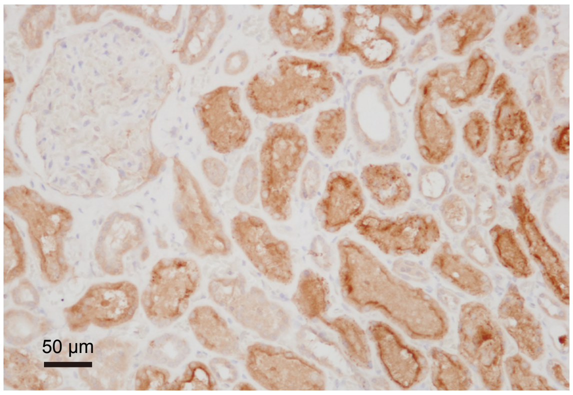

Aquaporin-1

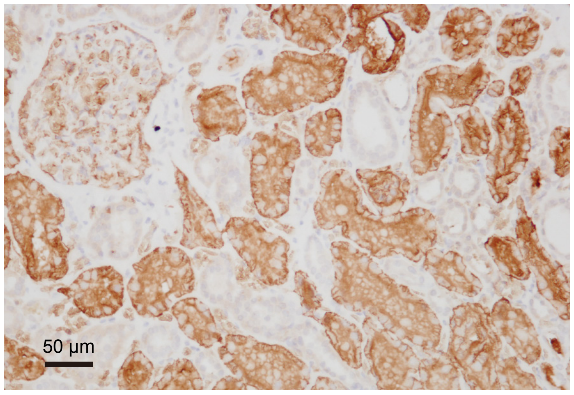

Supplementary Figure 3

Supplementary Figure 3. Immunochemical detection of ENPP6 in mouse kidney.
Kidney from adult mice was fixed and immunostained with anti-ENPP6 and anti-Aquaporin1 antibodies.

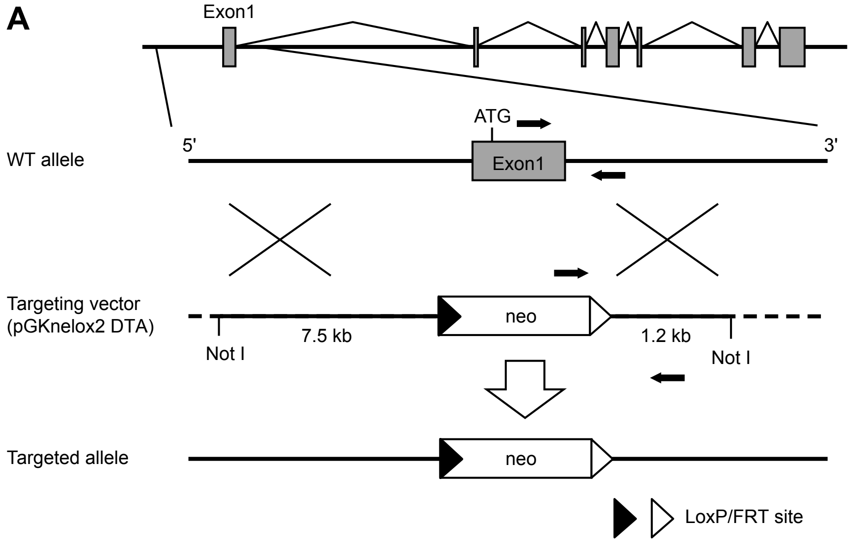

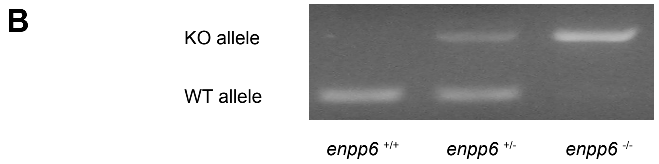

Supplementary Figure 4

Supplementary Figure 4. Construction of the ENPP6 KO mice by homologous recombination.

(A) Targeting strategy showing the structures of the wild-type *ENPP6* gene (top and second), the targeting vector (third) and the targeted allele (bottom). Filled rectangles indicate exons, whereas open rectangles show the neomycin resistance gene cassette. Open and filled triangles indicate the position of LoxP/FRT sites. The arrows indicate the position of PCR primers used to detect both wild-type and mutant alleles. (B) Results of genotyping by PCR.

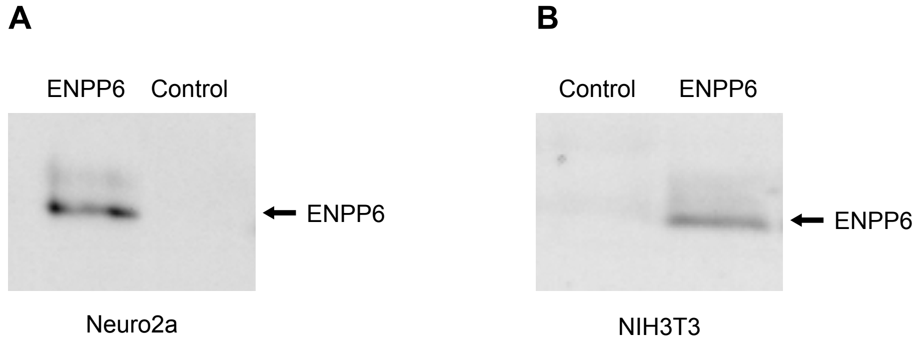

Supplementary Figure 5

Supplementary Figure 5. Stable expression of ENPP6 in Neuro2a and NIH3T3 cells.

Expression of ENPP6 protein in Neuro2a (A) and NIH3T3 (B) was examined by Western blot analysis.

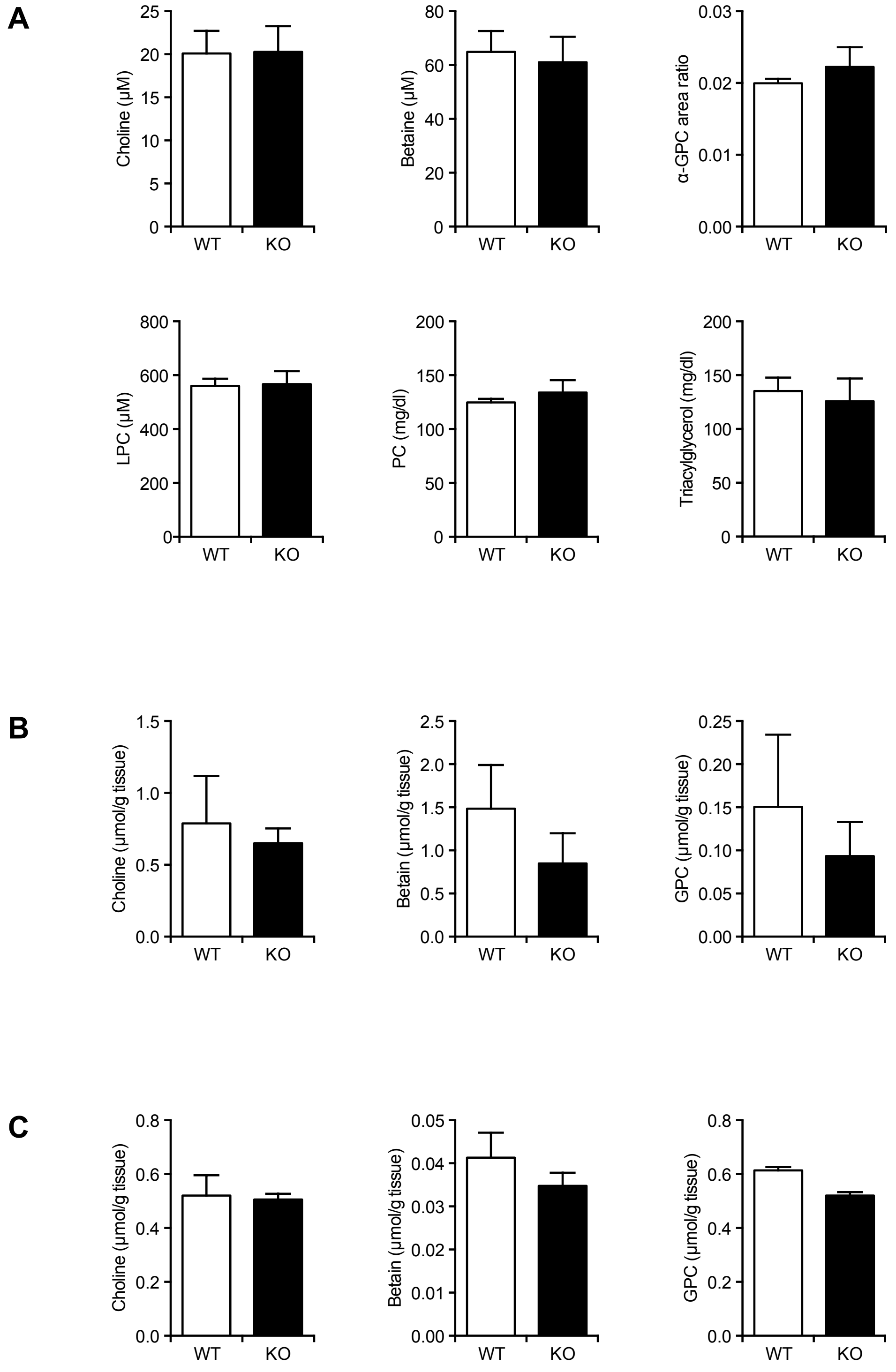

Supplementary Figure 6

Supplementary Figure 6. The levels of choline-containing compounds in ENPP6 KO mice.

Plasma and tissues were corrected from adult wild-type mice and ENPP6 KO mice. Then choline-containing compounds in plasma (A), liver (B) and brain (C) were extracted and determined using LC-MS/MS.

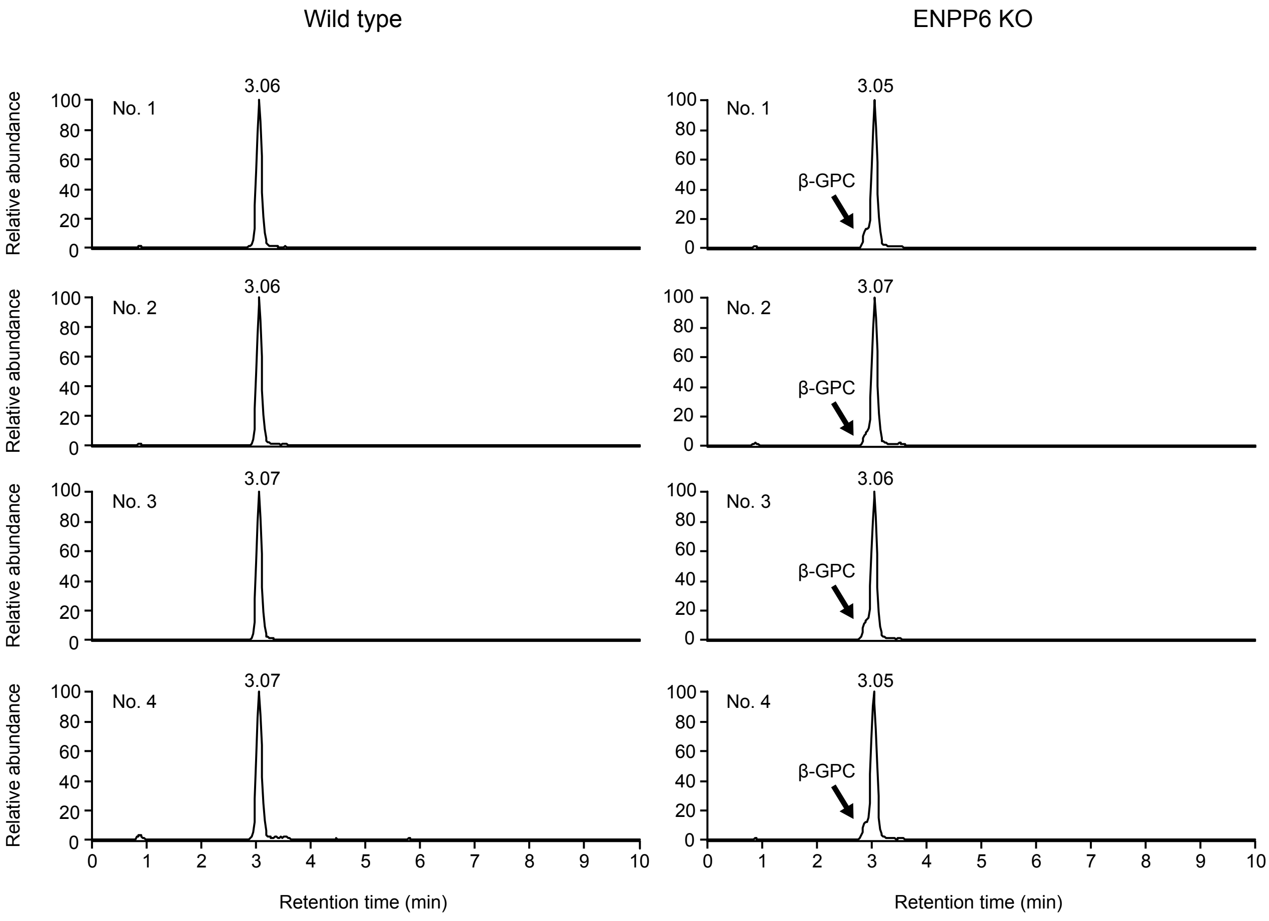

Supplementary Figure 7

Supplementary Figure 7. Detection of an α-GPC-like compound with *m/z* 257.9 in urine of ENPP6 KO mice by LC-MS/MS.

Mass chromatogram of α-GPC on reversed phased column chromatography. GPC with identical retention time with standard α-GPC was detected in the urine from wild-type mice, while a slightly faster migrating peak with identical *m/z* value was detected in the urine of ENPP6 KO mice (arrows).

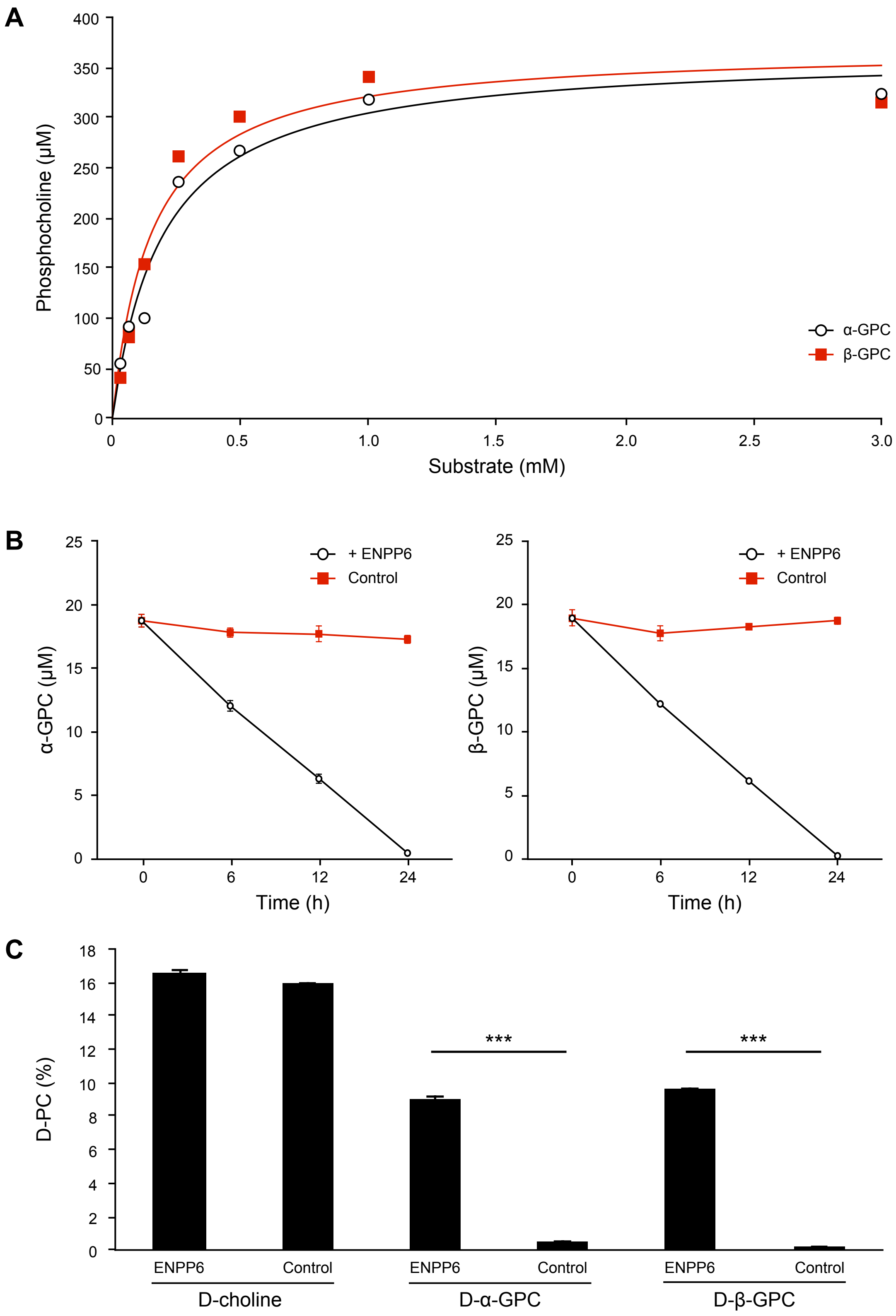

Supplementary Figure 8

Supplementary Figure 8. Biochemical characterization of β-GPC as a substrate of ENPP6.

(A) A kinetic analysis of ENPP6 showing that α- and β-GPC are similar as substrates for ENPP6. (B) α- and β-GPC were added to the culture media of ENPP6-expressing hepatoma cells and the level of remaining α- and β-GPC was determined by LC-MS/MS. (C) 24 h after the addition of D-α- and D-β-GPC, cells were recovered and the level of D-PC was determined by LC-MS/MS.

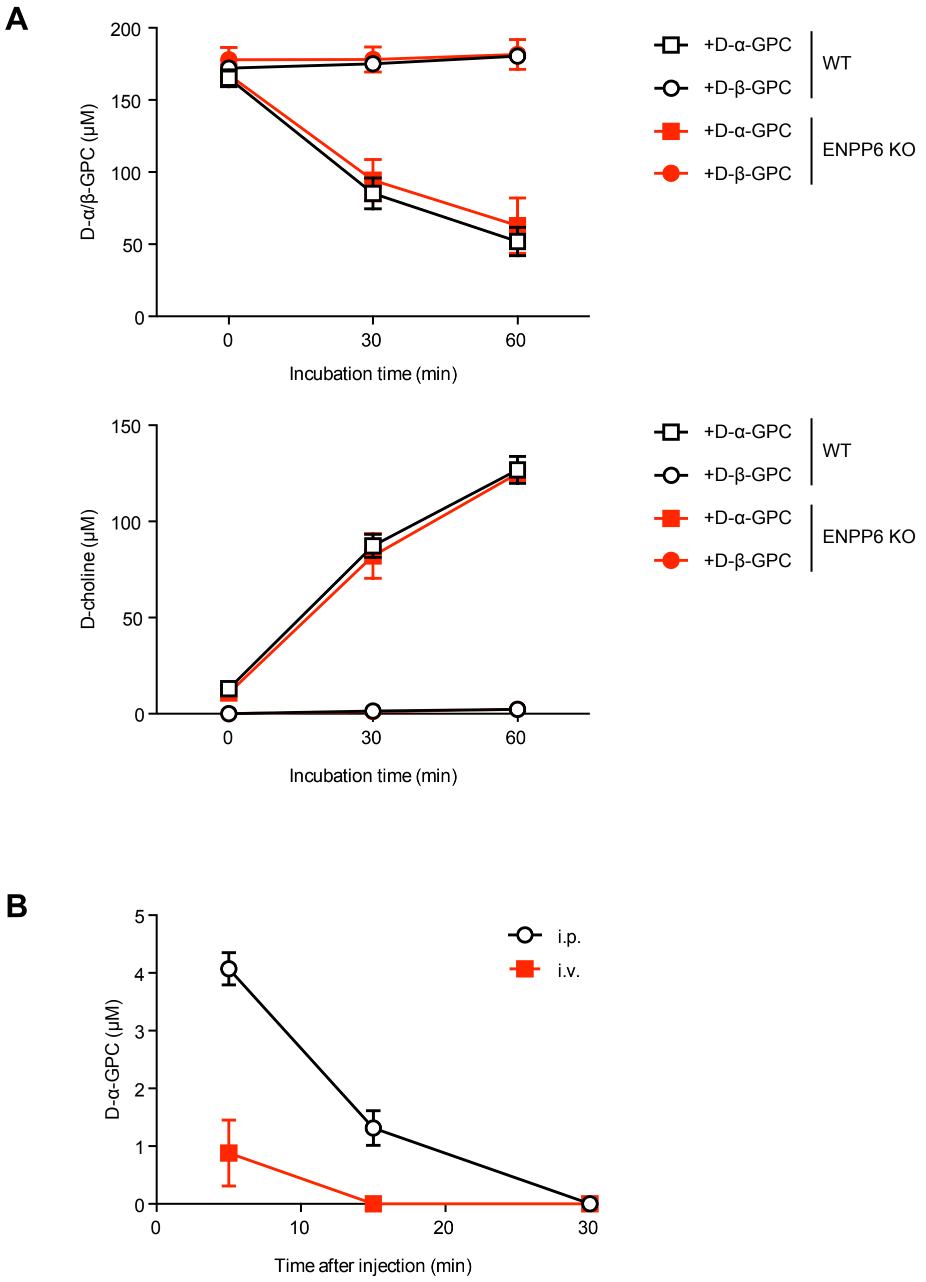

Supplementary Figure 9

Supplementary Figure 9. Stability of α-GPC and β-GPC.

(A) Stability of α-GPC and β-GPC in isolated whole blood. D-α-GPC and D-β-GPC were mixed with whole mouse blood isolated from wild-type or ENPP6 KO mice, and time-dependent degradation of both GPCs and production of D-choline were determined by LC-MS/MS. The analysis revealed that β-GPC is much more stable than α-GPC at least in whole blood, and indicated the presence of an ENPP6-independent degradation pathway of α-GPC. (B) Sustained plasma α-GPC level in mice injected with α-GPC via *i.p.*. Mice were injected with D-α-GPC via *i.v.* or *i.p.* At indicated time, plasma was collected and the remaining α-GPC level was determined by LC-MS/MS.

A

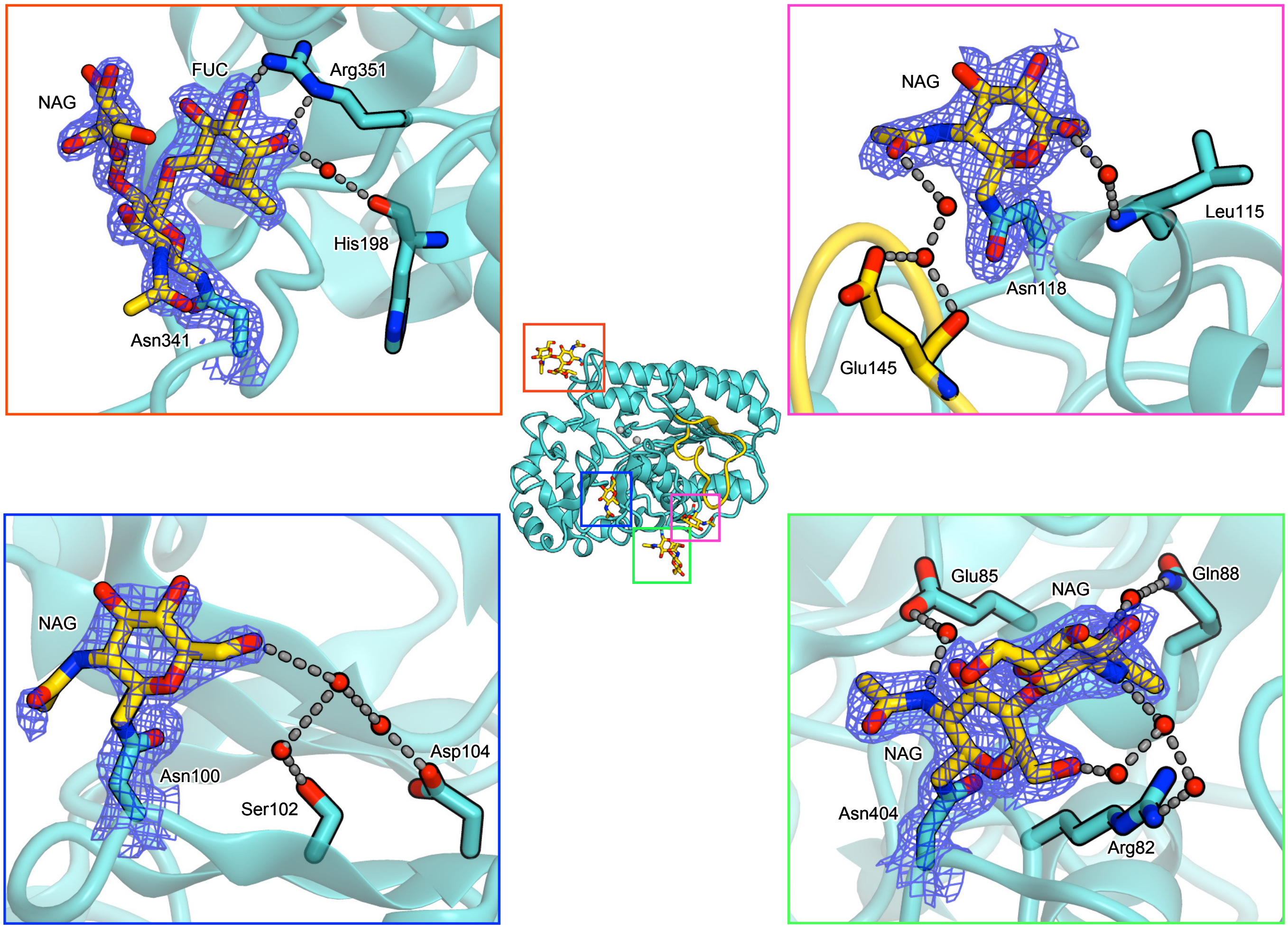

B

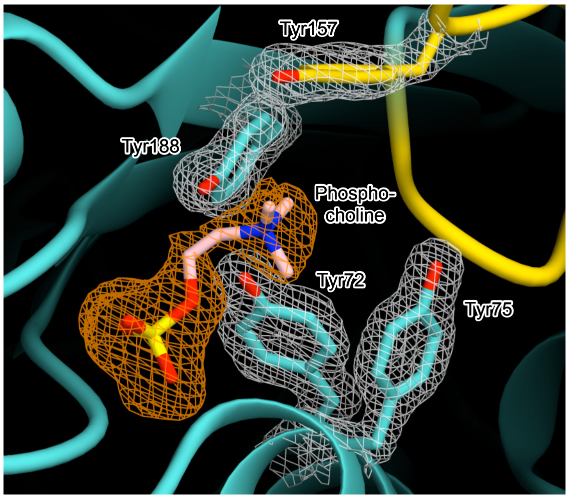

Supplementary Figure 10

Supplementary Figure 10. Electron density maps of ENPP6.

(A) *N*-linked glycans of apo ENPP6. $2F_O - F_C$ electron density maps of *N*-linked glycans (contoured at 1σ) are shown in blue meshes. Water molecules are shown as red spheres. The *N*-acetylglucosamine (NAG) residues of the glycans linked to Asn100, Asn118 and Asn404 form water-mediated hydrogen bonds with the protein, and a fucose (FUC) residue of the Asn341-linked glycan forms direct and water-mediated hydrogen bonds with Arg351 and His198, respectively. Point mutations of these asparagine residues resulted in markedly reduced expression of ENPP6 in the culture supernatants of HEK293T cells[1]. These observations indicated that the intramolecular interactions between the *N*-linked glycans and protein surface are important for the stability of ENPP6. (B) The active site of ENPP6. A simulated annealing $F_O - F_C$ omit electron density map (contoured at 3σ) of phosphocholine is shown in orange mesh, and a $2F_O - F_C$ electron density map (contoured at 2σ) of Tyr72, Tyr75, Tyr157 and Tyr188 is shown in white mesh.

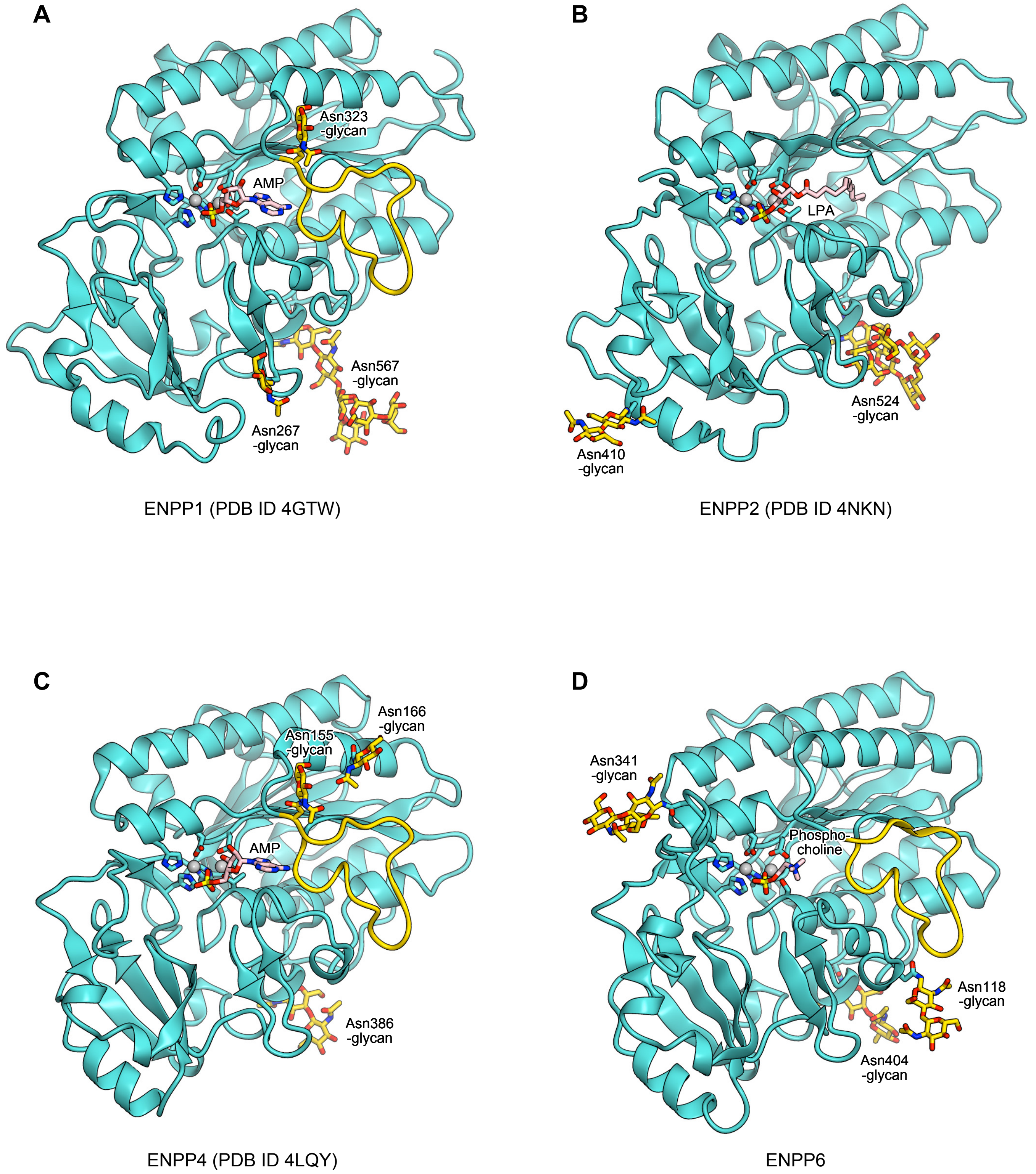

Supplementary Figure 11

Supplementary Figure 11. Structural comparison of the PDE catalytic domains of ENPP1, ENPP2, ENPP4 and ENPP6.

(A–D) Crystal structures of the PDE domains of ENPP1 in complex with AMP (PDB ID 4GTW) (A), ENPP2 in complex with 14:0-LPA (PDB ID 3NKM) (B), ENPP4 in complex with AMP (PDB ID 4LQY) (C), and ENPP6 in complex with phosphocholine (D). The bound zinc ions are shown as gray spheres, and the active-site residues are shown as sticks. *N*-linked glycans are shown as gold sticks. The "insertion" loops are highlighted in gold.

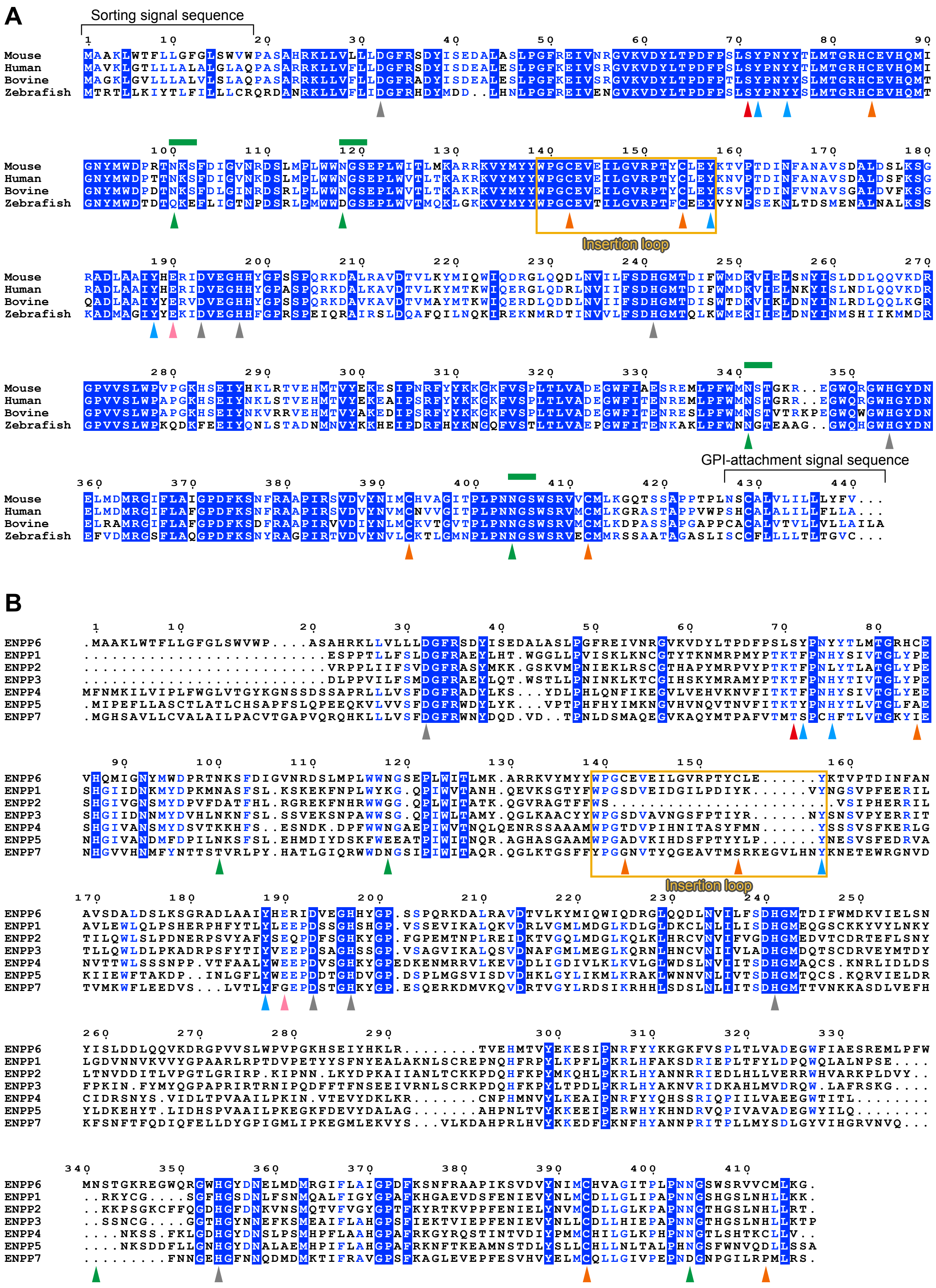

Supplementary Figure 12

Supplementary Figure 12. Multiple amino acid sequence alignment.

(A) Multiple sequence alignment of ENPP6 from different animal species. (B) Multiple sequence alignment of the mouse ENPP family proteins. The zinc-coordinating residues, gray triangles; the catalytic residue, red triangles; the tyrosine residues in the choline-binding pocket, light blue triangles; the residues hydrogen bonding with the tyrosine residues in the choline-binding pocket, light pink triangles; cysteine residues, orange triangles; the *N*-glycosylated asparagine residues, green triangles; the consensus sequences for *N*-glycosylation, green lines; the residues in the insertion loop, orange boxes.

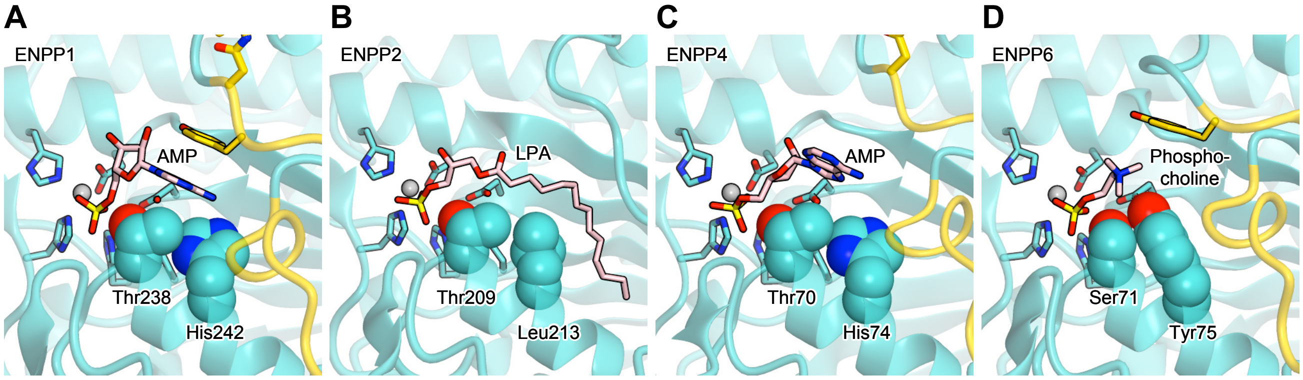

Supplementary Figure 13

Supplementary Figure 13. Catalytic nucleophiles of the ENPP family members. Active sites of ENPP1 in complex with AMP (PDB ID 4GTW) (A), ENPP2 in complex with 14:0-LPA (PDB ID 3NKM) (B), ENPP4 in complex with AMP (PDB ID 4LQY) (C), and ENPP6 in complex with phosphocholine (D). Ser71 and Tyr75 of ENPP6 and corresponding residues in ENPP1, ENPP2 and ENPP4 are shown as space-filling models.

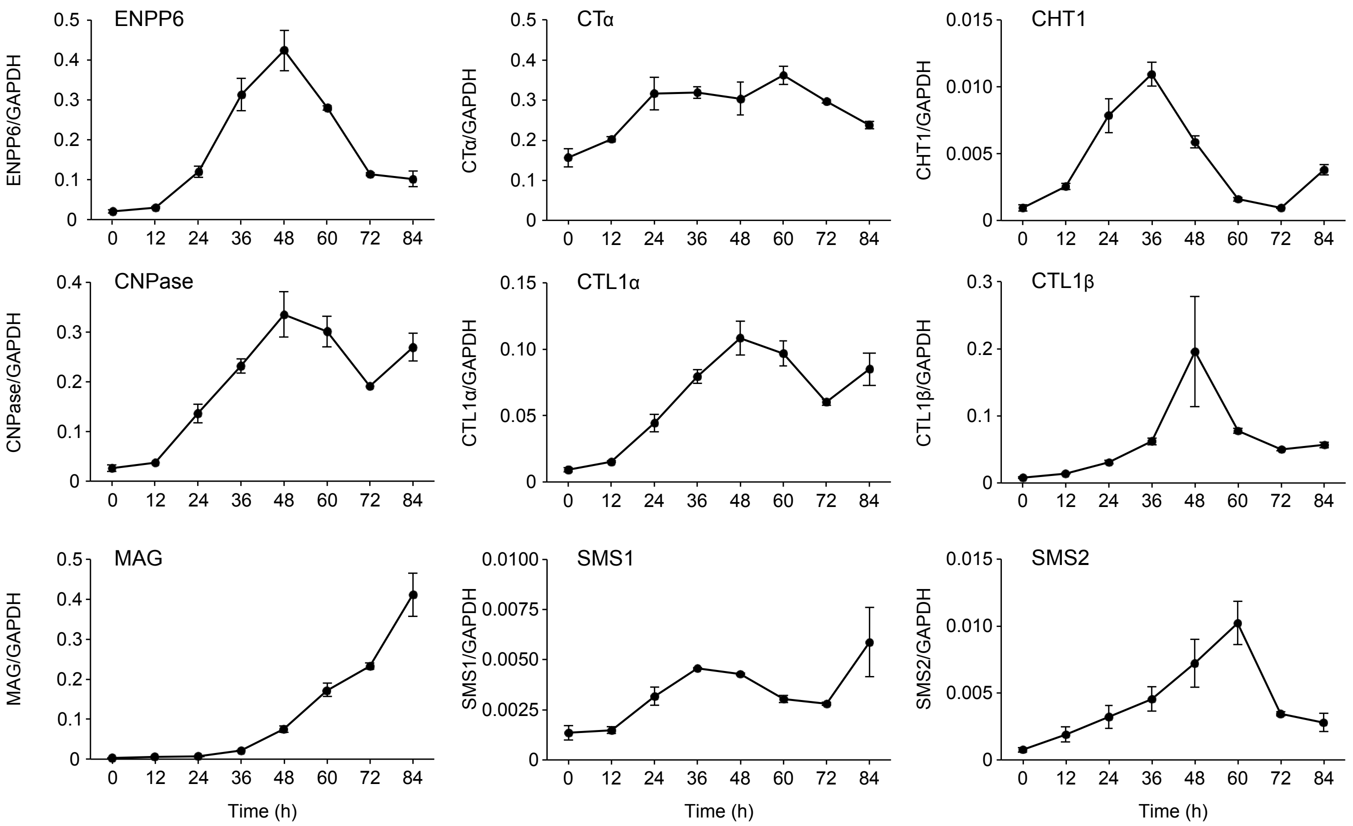

Supplementary Figure 14

Supplementary Figure 14. Expression of choline-related genes and oligodendrocyte differentiation genes during oligodendrocyte differentiation.

OPCs were treated with differentiating medium, and total mRNA was then collected 0, 12, 24, 36, 48, 60, 72 and 96 h after the induction of differentiation. Expression of choline-related genes including ENPP6 was examined by quantitative real time RT-PCR. We examined genes involved in choline transport (high-affinity choline transporter CHT1 and low-affinity choline transporters CTL1α and CTL1β), genes in choline metabolism (ENPP6, CTP:phosphocholine transferase CTα and sphingomyelin synthases SMS1 and SMS2), and markers for oligodendrocyte differentiation (2',3'-cyclic-nucleotide 3'-phosphodiesterase CNPase and Myelin-associated glycoprotein precursor MAG). GAPDH was used as an internal standard.

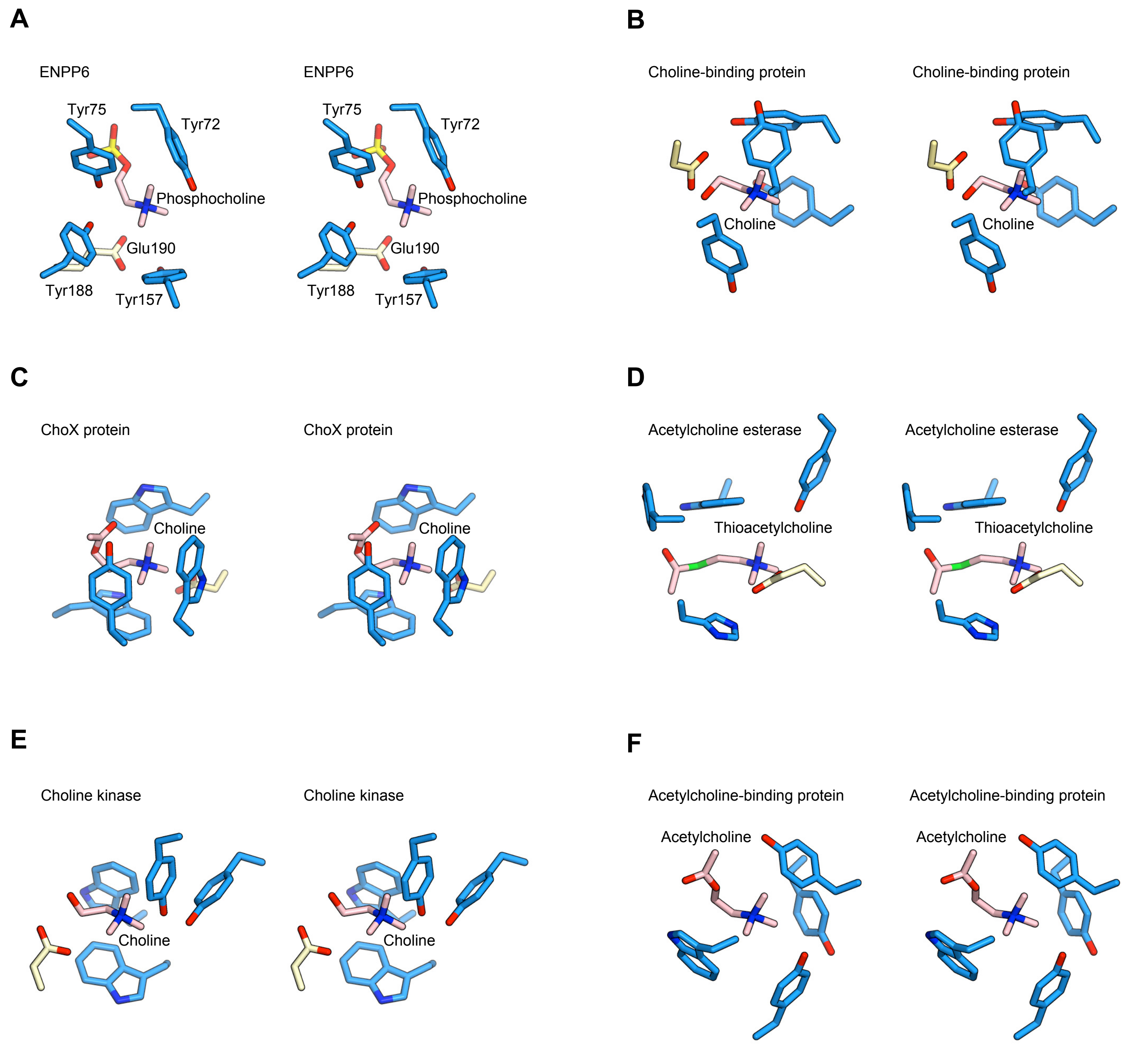

Supplementary Figure 15

Supplementary Figure 15. Choline recognition in various choline-binding proteins (stereo view).

(A) ENPP6. (B) Bacteria choline-binding protein (PDB ID 3R6U). (C) Bacteria ChoX (PDB ID 2RIN). (D) Electric ray acetylcholine esterase (PDB ID 2C4H). (E) Protist choline kinaes (PDB ID 3C5I). (F) Sea hare acetylcholine-binding protein (PDB ID 2XZ5). The residues in the choline-binding pocket and the choline-containing compounds are shown as blue and light pink sticks, respectively.

SUPPLEMENTAL TABLE

Table S1. Oligonucleotide primers for real-time RT-PCR

Target gene		Primer sequence
ENPP6	forward	GTAGTCATCTTGGACCCTCTCATACTG
	reverse	GTGTGAGCTCTTACATGTGGACAGA
GAPDH	forward	GCCAAGGTCATCCATGACAACT
	reverse	GAGGGGCCATCCACAGTCTT
CNPase	forward	GGGAAGGCCGCTGGG
	reverse	GTCACAAAGAGAGCAGAGATGGAC
MAG	forward	GAGCCACTGCCTTCAACCTG
	reverse	GCCGCTGCACAGTGTGACT
CTα	forward	TCTGCAGGGAGCGATGATG
	reverse	TGTGGAGATACCTTCTGTCCTCTG
CHT1	forward	GCTCTACCCGCCATATGCAT
	reverse	CCGTAGGCAGTCTGGTTC
SMS1	forward	TATCACACGATGGCCAATCAG
	reverse	CCAATGGTAAGATCGAGGTACAAT
SMS2	forward	CCGGAATGCACTTCCAGTGT
	reverse	AGCCGGAGAATCCGCTGTAT
CTL1α	forward	AGCCCTGGCCGAGAATTC
	reverse	TCATTGCTTTCCTACTGTTTTCCA
CTL1β	forward	AGCCCTGGCCGAGAATTC
	reverse	GCTCTCGGGCGTCCG

SUPPLEMENTAL EXPERIMENTAL PROCEDURES

Generation of ENPP6 knockout mice

Targeting vector was created by inserting 7.5 kb of 5' homologous region and 1.2-kb of 3' homologous region into PGKneolox2DTA vector. The targeting vector was introduced into 129/SvE ES cells by electroporation. Among four G418-resistant ES clones confirmed for targeted homologous recombination by Southern blot analysis, two independent clones were used for blastocyst injection. Heterozygous (*ENPP6*$^{+/-}$) mice were derived from subsequent germline transmission of chimeric male mice. Heterozygous mice were backcrossed to C57BL6/J mice (CLEA Japan) 8 times. Homozygous (*ENPP6*$^{-/-}$) mice were obtained from intercross of heterozygotes. In all experiment using homozygous mice, littermates were used for control.

Monoclonal antibody to mouse ENPP6

cDNA for the extracellular domain of mouse ENPP6 (residues 23–419) was amplified by PCR using mouse kidney cDNA as a template DNA and two oligonucleotide primers, 5'-GCCGGC*CCCGGG*CCACCGGAAGCTCCTGGTGTTGC-3' and 5'-GGTGGG*CTCGAG*TTAGCTGGTCTGGCCCTTCAGCATG-3'. The resulting DNA fragment was introduced into the baculovirus transfer vector pFASTBac-1 (Invitrogen, Carlsbad, CA), which was modified to have a polyhistidine-tag added at the NH-terminus[2]. The preparation of recombinant baculovirus and the expression of recombinant mouse ENPP6 were performed according to the manufacturer's protocol. After the nucleotide sequence was confirmed, the recombinant baculovirus was prepared using Bac-to-Bac Baculovirus Expression system (Invitrogen). After recombinant soluble ENPP6 protein was expressed in Sf9 cells by infecting the virus for 96 hours at a M.O.I. of 10, it was purified using Nickel column chromatography (GE healthcare, Piscataway, NJ). *WYK/Izm* rats were immunized with the recombinant protein (50 µg/rat) with Freund's complete adjuvant. The enlarged medial iliac lymph nodes from the immunized rats were used for cell fusion with mouse myeloma cells, PAI. The antibody-producing hybridoma cells were screened by ELISA, immunofluorescence and Western blotting. In this study we established four hybridoma cell lines and used clone 10B10 for Western blotting and immunofluorescence analyses.

Stable ENPP6-expressing rat hepatoma

Mouse NPP6 and NPP6 mutant cDNA were introduced into the retrovirus vector pMXs-IG[3]. Obtained vector was transfected into PLAT-E cells[4]. PLAT-E cells were kindly gifted by Dr. Kitamura. Ten ml of culture supernatants of PLAT-E cells containing the viruses were collected and filtered through a 0.45 μm filter. For infection, the virus stock was added into Rh7777 cells with polybrene (Sigma, St. Louis, MO). After 2 times infection, GFP positive cells were sorted by Cell Sorter SH800 (Sony, Tokyo, Japan).

Oligodendrocyte progenitor cell (OPC) culture

Mouse oligodendrocyte progenitor cells (OPCs) cultures were established by modified method of Yang[5] and Seiwa[6]. Briefly, cortexes from E15 mice were dissociated by dispase and DNaseI, and the cells were cultured on poly-D-lysine coated T-75 flask in MEM-E medium supplemented with 10% fetal bovine serum (FBS), 0.5% glucose and antibiotics. After five days, the flasks were shaken at 450 rpm for 5 min. This operation detaches the microglial cells from the bottom of the flasks. Then, the microglial cells were removed by replacing the media. After incubating the cells for another 2 hours, the flasks were shaken overnight at 200 rpm to dislodge OPCs from feeding monolayer cells. The resulting supernatant containing OPCs was transferred to a new Petri dish and cultured for 30 min to remove astrocytes. Through these operations, less adhesive OPCs were recovered from the supernatant. Then, the supernatant was transferred to a new culture dish coated with poly-L-lysine. The basic medium for OPCs is modified B-S medium (DMEM containing 0.66% glucose, 0.01% BSA, 5 μg/ml insulin, 0.5 μg/ml transferrin, 0.06 μg/ml progesterone, 16 μg/ml putrescine, 40 ng/ml sodium selenate and antibiotics) supplemented with 0.4% ASF104 (Ajinomoto, Tokyo Japan), 20 ng/ml PDGF, 10 ng/ml bFGF and 5% Nerve-Cell Culture Medium (MB-X9501, Sumitomo Bakelite, Akita, Japan). The half of medium was exchanged every two days. OPCs were cultured for 5-6 days until they reach to 80% confluency. The cells were then treated with 0.25% trypsin and plated with optimal cell density for each experiment. We confirmed that after these procedures, >95% cells were oligodendrocyte lineage as judged by NG2 staining. To differentiate OPCs to oligodendrocytes, OPCs were cultured in differentiating medium (modified B-S medium supplemented with 40 ng/ml

thyroxine, 30 ng/ml triiodothyronine and 20% Nerve-Cell Culture Medium) for indicated time. To examine choline requirement for proliferation of OPCs and oligodendrocytes, we used choline-deficient differentiating medium (choline-deficient modified B-S medium supplemented with 40 ng/ml thyroxine, 30 ng/ml triiodothyronine and 20% choline deficient Nerve-Cell Culture Medium). Choline chloride-deficient DMEM (Cell Science & Technology Institute, Sendai, Japan) was used as the choline-deficient B-S medium. Choline deficient Nerve-Cell Culture Medium were prepared by removing components with low molecular weight (<10 kDa) using centrifugal filter units (Amicon Ultra, Millipore, Billerica, MA).

Quantification of choline containing compounds and phospholipids by LC-MS/MS

Our liquid chromatography–mass spectroscopy (LC-MS/MS) system consisted of a NANOSPACE SI-2 HPLC (Shiseido Co., Ltd., Tokyo, Japan) and a TSQ Quantum Ultra triple quadropole mass spectrometer (Thermo Fisher Scientific, Waltham, MA, USA) equipped with a heated ESI source. For analysis of choline-containing compounds and lipids, they were extracted from cells (1×10^5) by suspending cells in 100 μl methanol containing internal standards 17:0 LPC or 14:0/14:0 PC. Choline-containing compounds were also extracted from tissues (< 50 mg) in 1 ml methanol containing internal standards. The extracts were subjected to sequential centrifugation, and the resulting supernatants were analyzed after filtration.

For analysis of GPC, choline, betaine and their deuterated derivative, they were separated by a X-Briddge HILIC column (2.1 mm × 100 mm i.d., 3.5 μm particle size, Waters), using a gradient of solvent A (5 mM ammonium formate in 95% (v/v) water, pH 4.0) and solvent B (5 mM ammonium formate in 95% (v/v) acetonitrile, pH 4.0). Gradient conditions were as follows: hold 81.5% B for 1.6 min, a 0.9 min gradient to 20% B, hold 20% B for 4.5 min, return to the initial condition and hold for 3 min. The flow rate was set at 400 μl/min. For analysis of LPC, lipid was separated by a CAPCELL PAK C-18 column (1.5 mm × 250 mm i.d., 3 μm particle size, Shiseido Co., Ltd.), using same solvent as GPC analysis. Gradient conditions were as follows: hold 50% B for 0.2 min, a 2.5 min gradient to 95% B, hold 95% B for 2.5 min, return to the initial condition and hold for 1 min. The flow rate was set at 250 μl/min. For analysis of

PC and D-PC, they were separated by a SILLICA SG80 column (2.0 mm × 150 mm i.d., 5 μm particle size, Shiseido Co., Ltd.) using a gradient of solvent A (5 mM ammonium formate in water) and solvent B (acetonitrile). The initial condition was set at 100% B. The following solvent gradient was applied: a 15-min gradient to 82.5% B, a 2-min gradient to 30% B, a 2-min gradient to 100% B and hold for 4 min. The flow rate was set at 400 μl/min. All compounds were analyzed as $[M+H]^+$ in the positive ion mode. The product ions were as follows: m/z 60.2 (choline), m/z 69.2 (D-choline), m/z 104.2 (α-GPC), m/z 113.2 (D-α-GPC), m/z 184.0 (PC and LPC) and m/z 193 (D-PC). The ratio between analyte and internal standard peak area was used for quantification.

Chemical synthesis of α-GPC and β-GPC

Materials were obtained from commercial suppliers and used without further purification unless otherwise mentioned. Anhydrous CH_2Cl_2 was purchased from Kanto Chemical Co. Inc. Anhydrous Et_3N was dried and distilled according to the standard protocols. Flash column chromatography was performed on Silica Gel 60N (Kanto, spherical neutral, 40–50 μm). Analytical TLC was performed on Merck 60 F_{254} glass plates precoated with a 0.25 mm thickness of silica gel. NMR spectra were recorded on a JNM-AL400 spectrometer. Chemical shifts for ^{1}H-NMR are reported in parts per million (ppm) downfield from tetramethylsilane as the internal standard and coupling constants are in Hertz (Hz). The following abbreviations are used for spin multiplicity: s = singlet, d = doublet, t = triplet, q = quartet, m = multiplet, and br = broad. Chemical shifts for ^{13}C-NMR are reported in ppm, relative to the central line of a triplet at 77.0 ppm for deuteriochloroform. Melting points were determined on a Yanaco micro melting point apparatus and uncorrected. IR spectra were measured on a JASCO FT/IR-4100 spectorometer. Mass spectra were recorded on a Brucker micrOTOF II (ESI). Optical rotations were measured on a Horiba SEPA-300 high sensitive polarimeter.

L-α-Glycerophosphocholine-D$_9$

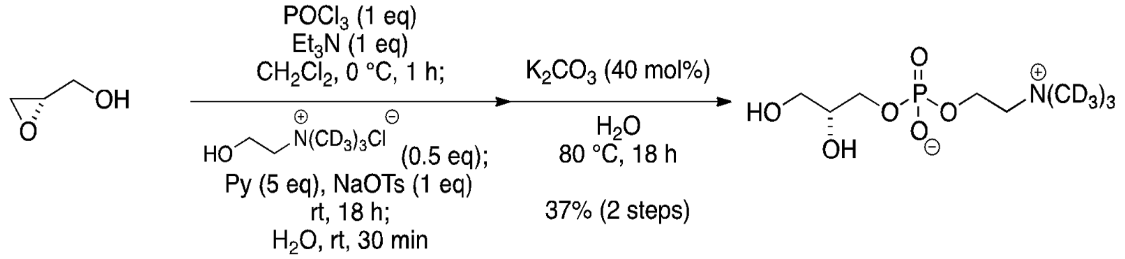

A 30-ml round-bottomed flask equipped with a magnetic stirring bar and straight inlet adapter with a side arm three-way stopcock was charged with $POCl_3$ (0.126 ml, 1.35 mmol) and CH_2Cl_2 (10 ml). To the solution was added dropwise a solution of *S*-(–)-glycidol (90.1 µl, 1.35 mmol) and Et_3N (0.188 ml, 1.35 mmol) in CH_2Cl_2 (10 ml) at 0 °C. After stirring for one hour at the same temperature, choline chloride (trimethyl-d_9) (100 mg, 0.672 mmol), pyridine (0.544 ml, 6.75 mmol), and sodium tosylate (272 mg, 1.40 mmol) were added to the resulting solution. After stirring for 18 hours at room temperature, H_2O (three drops) was added to the resulting suspension and the mixture was stirred for further 30 min at the same temperature. The reaction mixture was concentrated in vacuo, and the residue was purified by short-column chromatography on silica gel (CH_2Cl_2/MeOH/H_2O, 7:6:1 then 2:3:1 (v/v)). The crude material was subjected to next reaction without further purification.

A screw threaded glass tube equipped with a magnetic stirring bar was charged with the above residue, K_2CO_3 (40.0 mg, 0.289 mmol), and H_2O (2 ml) and caped with a teflon coated screw cap. After stirring for 18 h at 80 °C, the reaction mixture was neutralized with 1 M aqueous HCl solution and concentrated in vacuo. The residue was purified by flash column chromatography on silica gel (CH_2Cl_2/MeOH/H_2O, 2:3:1 (v/v)) to give α-glycerophosphocholine-d_9 (66.6 mg, 0.250 mmol, 37% for 2 steps) as a colorless gummy oil; $[\alpha]_D^{27}$ –2.2 (*c* 1.0, MeOH); IR (KBr) 3417, 2931, 1651, 1454, 1220, 777 cm^{-1}; ^1H NMR (400 MHz, CD_3OD) δ 4.33–4.26 (2H, m), 3.94 (1H, ddd, J = 10.8, 6.4, 4.4 Hz), 3.87 (1H, ddd, J = 10.8, 6.4, 4.4 Hz), 3.77 (1H, dt, J = 5.2, 5.2 Hz), 3.65–3.50 (4H, m); ^{13}C NMR (100 MHz, CD_3OD) δ 72.53, 72.46, 67.93, 67.87, 67.2, 63.8, 60.41, 60.36; LRMS (ESI) *m/z* 267 $[M+H^+]$; HRMS (ESI) *m/z* Calcd for $C_8H_{12}D_9NO_6P$ 267.1666 $[M+H^+]$, Found 267.1661.

1,3-B 2-glycerophosphocholine-D$_9$

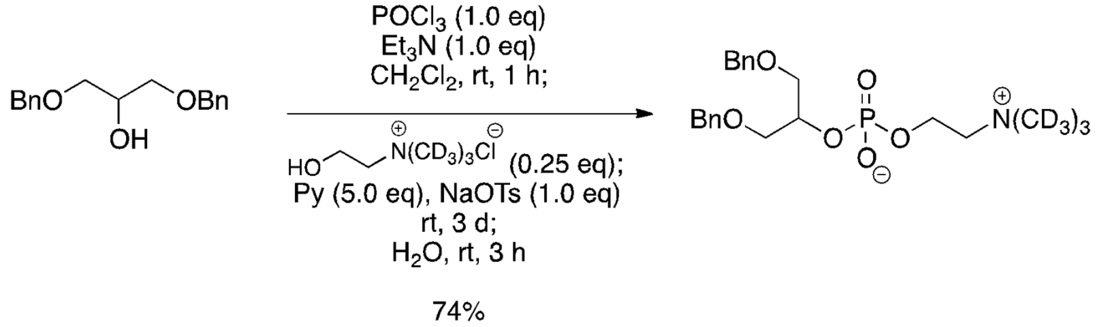

A 200-ml round-bottomed flask equipped with a magnetic stirring bar and a straight inlet adapter with a side arm three-way stopcock was charged with $POCl_3$ (1.37 ml, 14.7 mmol) and CH_2Cl_2 (5.0 ml). To the solution was added dropwise a solution of 1,3-bisbenzylglycerol (4.01 g, 14.7 mmol) and Et_3N (2.82 ml, 20.2 mmol) in CH_2Cl_2 (15 ml) at 0 °C. After stirring for one hour at room temperature, choline chloride (trimethyl-d_9) (500 mg, 3.36 mmol), pyridine (5.42 ml, 67.3 mmol), and sodium tosylate (2.80 g, 14.4 mmol) were added to the resulting solution. After stirring for three days at room temperature, H_2O (5.0 ml) was added to the resulting suspension and the resulting mixture was stirred for further three hours at the same temperature. The reaction mixture was concentrated in vacuo, and the residue was purified by MPLC on silica gel (CH_2Cl_2/MeOH/H_2O, 4:6:1 to 2:3:1 (v/v)) to give phosphodiester (1.09 g, 2.43 mmol, 74%) as a pale yellow oil; IR (neat) 3376, 1653, 1459, 1229, 1079, 949 cm^{-1}; ^1H NMR (400 MHz, $CDCl_3$) δ 7.33–7.26 (10H, m), 4.52–4.43 (5H, m), 4.18–4.08 (2H, m), 3.70 (4H, d, J = 5.2 Hz), 3.42–3.35 (2H, m); ^{13}C NMR (100 MHz, $CDCl_3$) δ 138.3, 128.2, 127.7, 127.4, 77.2, 73.0, 72.9, 72.8, 70.8, 70.7, 65.6, 65.5, 58.9, 58.8; LRMS (ESI) *m/z* 447 $[M+H^+]$; HRMS (ESI) *m/z* Calcd for $C_{22}H_{24}D_9NO_6P$ 447.2605 $[M+H^+]$, Found 447.2606.

***β*-Glycerophosphocholine-*D*$_9$**

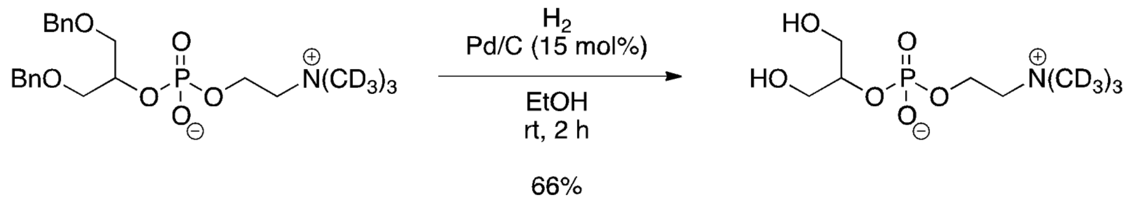

A 30-ml round-bottomed flask equipped with a magnetic stirring bar and a straight inlet adapter with a side arm three-way stopcock was charged with phosphodiester (1.08

g, 2.42 mmol), 10% Pd/C (382 mg, 0.359 mmol), and EtOH (15 ml). After stirring under hydrogen atmosphere for 2 h at room temperature, the resulting suspension was filtered through a pad of Celite and the filter cake was washed with MeOH. After removal of solvent under reduced pressure, the residue was purified by short-column chromatography on silica gel (MeOH/H_2O, 7:3 (v/v)) to give a white solid, which was recrystallized from MeOH–EtOAc to afford *β*-glycerophosphocholine-d_9 (423 mg, 1.59 mmol, 66%) as colorless needles. Mp 202–203°C (MeOH–EtOAc); IR (ATR) 3220, 1324, 1065, 966, 944, 788 cm^{-1}; ^{1}H NMR (400 MHz, CD_3OD) δ 4.39–4.25 (2H, m), 4.20–4.16 (1H, m), 3.74–3.55 (6H, m); ^{13}C NMR (100 MHz, CD_3OD) δ 79.22, 79.16, 67.3, 63.5, 63.4, 60.5, 60.4; LRMS (ESI) *m/z* 267 [$M+H^+$]; HRMS (ESI) *m/z* Calcd for $C_8H_{12}D_9NO_6P$ 267.1666 [$M+H^+$], Found 267.1669.

SUPPLEMENTAL REFERENCES

1 Vagin, A. & Teplyakov, A. Molecular replacement with MOLREP. *Acta Crystallogr D Biol Crystallogr* **66**, 22-25 (2010).

2 Tsuda, S. *et al.* Cyclic phosphatidic acid is produced by autotaxin in blood. *J Biol Chem* **281**, 26081-26088 (2006).

3 Kitamura, T. *et al.* Retrovirus-mediated gene transfer and expression cloning: powerful tools in functional genomics. *Exp Hematol* **31**, 1007-1014 (2003).

4 Morita, S., Kojima, T. & Kitamura, T. Plat-E: an efficient and stable system for transient packaging of retroviruses. *Gene Ther* **7**, 1063-1066 (2000).

5 Yang, Z., Watanabe, M. & Nishiyama, A. Optimization of oligodendrocyte progenitor cell culture method for enhanced survival. *J Neurosci Methods* **149**, 50-56 (2005).

6 Seiwa, C. *et al.* Bisphenol A exerts thyroid-hormone-like effects on mouse oligodendrocyte precursor cells. *Neuroendocrinology* **80**, 21-30 (2004).
